# Coffee and tea consumption and cardiovascular disease and all-cause and cause-specific mortality in individuals with diabetes mellitus: a meta-analysis of prospective observational studies

**DOI:** 10.3389/fnut.2025.1570644

**Published:** 2025-06-02

**Authors:** Li Ding, Hai-Peng Wang, Jun-Yu Zhao, Xin Zhao, Yu Sha, Li-Qiang Qin, Khemayanto Hidayat

**Affiliations:** ^1^Department of Nutrition and Food Hygiene, School of Public Health, Soochow University, Suzhou, China; ^2^Department of Cardiovascular, The First Affiliated Hospital of Soochow University, Suzhou, Jiangsu, China; ^3^School of Medicine, Jiangsu University, Zhenjiang, China; ^4^Department of Medical Technology, Suzhou Vocational Health College, Suzhou, China

**Keywords:** coffee, tea, mortality, CVD, meta-analysis, dose-response, diabetes mellitus

## Abstract

**Background:**

Individuals with diabetes mellitus (DM) are more likely to develop cardiovascular disease (CVD) and die prematurely than those without this condition. Coffee or tea consumption has been linked with lower risks of developing CVD and premature death in general populations. A meta-analysis of published prospective observational studies was performed to provide up-to-date evidence on the association between tea or coffee consumption and the risks of CVD and all-cause and cause-specific mortality in individuals with DM.

**Methods:**

The PubMed, Web of Science, Cochrane Library and Embase databases were searched. A Random-effects model was used to estimate the pooled hazard ratios (HRs) and 95% confidence intervals (CIs).

**Results:**

Compared with the lowest consumption category, the highest coffee consumption was associated with lower risks of all-cause mortality (HR 0.82, 95%CI 0.73, 0.91; *n* = 9), coronary heart disease (CHD) mortality (HR = 0.66, 95% CI: 0.51, 0.85; *n* = 3), CVD incidence (HR = 0.85, 95% CI: 0.75, 0.97; *n* = 2), and CHD incidence (HR = 0.82, 95% CI: 0.68, 0.99; *n* = 3). Similarly, the highest tea consumption was associated with lower risks of all-cause mortality (HR = 0.85, 95%CI: 0.79, 0.92; *n* = 6) and CVD mortality (HR = 0.86, 95% CI: 0.80, 0.93; *n* = 5). Linear associations were observed between coffee consumption and the risks of CVD mortality, CHD mortality, CVD incidence, and CHD incidence, as well as between tea consumption and the risk of CVD mortality. Nonlinear associations were found between coffee or tea consumption with the risk of all-cause mortality, with the greatest risk reduction observed at one to four cups of coffee per day or up to two cups of tea per day. The certainty of the evidence was mostly graded as moderate for coffee consumption (except for cancer mortality and stroke incidence, which were graded as low) and low for tea consumption (except for CVD mortality, which was graded as moderate).

**Conclusion:**

Daily coffee or tea consumption may be associated with lower risks of CVD and death, particularly from CVD, among individuals with DM. However, However, due to the observational design, methodological limitations, and low to moderate certainty, these findings should be interpreted cautiously.

## Introduction

Diabetes mellitus (DM) is a life-long, chronic disease characterized by chronic hyperglycemia. Individuals with DM are at increased risk of developing cardiovascular disease (CVD) and dying prematurely from all or specific causes (1–4). Over the last few decades, there has been an increasing number of individuals living with DM [90% of which are type 2 DM (T2DM)] worldwide, and this trend is expected to continue in the next decades (5), creating a major global public health issue due to its related complications and death. Emerging evidence suggests that T2DM, which makes up the vast majority of DM cases, is largely preventable through lifestyle modification such as adopting a healthy diet, maintaining a healthy body weight, being physically active, being sober, and not smoking. Lifestyle modification is also one of the important factors in managing DM and reducing its cardiovascular and other complications (6–8).

Tea and coffee are among the most commonly consumed beverages in the world and contain antioxidants and other biologically active compounds that may benefit health (9–12). Since tea (13–15) or coffee (16, 17) consumption has been linked to lower risks of death from all-cause and CVD and several chronic diseases (e.g., CVD, T2DM, and certain types of cancer) in general populations, it would be of interest to further evaluate whether such cardioprotection and longevity are also seen in individuals with DM who are at increased risk of CVD and death from all or specific causes. Thus far, prospective observational studies (18–35) on the association between tea (18, 28, 29, 31, 32, 34) or coffee (19–27, 30–33, 35, 36) consumption and the risks of CVD death from all or specific causes in patients with DM have yielded conflicting results. While the previous meta-analysis (37) of prospective observational studies has provided some indications of the potential role of coffee consumption in preventing CVD and premature death in patients with DM, several articles were not included, including one (30) missing article that was available at that time and four (31–34) recently published articles, three of which reported the updated findings from several studies (20, 21, 35). Moreover, no meta-analysis has been performed to clarify the unsettled association between tea consumption and the risks of CVD and all-cause and cause-specific mortality. Given all these considerations, a meta-analysis of published prospective observational studies was performed to provide up-to-date evidence on the association between tea or coffee consumption and the risks of CVD and all-cause and cause-specific mortality in individuals with DM.

## Methods

The present meta-analysis was prepared and reported according to the Preferred Reporting Items for Systematic Reviews and Meta-Analyses (PRISMA) statement (38). The research question was guided by the Participants, Interventions, Comparisons, Outcomes, and Study (PICOS) framework. Two investigators (L.D. and H.-P.W.) independently performed the literature search, study selection, data extraction, and assessments of the risk of bias (RoB) and the certainty of the evidence. Discrepancies between the two investigators were resolved by consensus.

### Literature search

We searched the PubMed, Web of Science, Embase, and Cochrane Library databases from inception to April 2025. The complete search strategy for each database is reported in Supplementary Table S1. Additionally, we checked the reference lists of the included studies to avoid missing relevant studies. Previous reviews and meta-analyses on the topic were also used as information sources. We did not contact relevant authors of the retrieved publications for additional information, as this approach rarely succeeds. There were no restrictions on language or publication date.

### Study selection

The PICOS framework is shown in Table 1. Briefly, prospective observational studies with either a prospective cohort, case-cohort, or nested case-control design were considered relevant if they reported risk estimates [relative risks (RRs), hazard ratios (HRs), or odds ratios (ORs)] and 95% confidence intervals (CIs) for the association between tea or coffee consumption and the risk of all-cause and cause-specific mortality and incidence of CVD in adults with DM. When multiple articles from the same study population were available, we included the one with the most up-to-date findings (i.e., the largest number of events and longest follow-up duration) in the main analysis. When multiple articles from the same study population were available, we included the one with the most up-to-date findings (i.e., the largest number of events and longest follow-up duration) in the main analysis. Overlapping articles were retained only if they provided unique data—such as sex-specific estimates, cause-specific mortality, or CVD subtypes—not available in the primary article. These data were used in subgroup analyses, where appropriate, or summarized descriptively when meta-analysis was not feasible. For studies with missing or incomplete data, we attempted to contact corresponding authors to obtain clarifications or additional details. If no response was received, such studies were excluded from the quantitative synthesis but considered for qualitative discussion when appropriate. In cases where key numerical data were partially reported (e.g., HRs without CIs), we used established statistical methods to approximate missing values, provided that reasonable assumptions could be made. These procedures were applied consistently to enhance transparency and reduce potential bias arising from incomplete reporting.

**Table 1:** Parameters: Participants, Interventions, Comparisons, Outcomes, and Study (PICOS) design framework.

	**Inclusion criteria**	**Exclusion criteria**
Participants	Adults with diabetes mellitus	Adults without diabetes mellitus
Intervention or exposure	Tea or coffee consumption	Caffeine consumption
Comparison	Highest vs. lowest category of tea or coffee consumption; per cup increase in daily coffee consumption	
Outcome	Risk estimates (relative risks, hazard ratios, or odds ratios) and 95% confidence intervals for cardiovascular disease incidence and all-cause and cause-specific mortality	Risk estimates (relative risks, hazard ratios, or odds ratios) and 95% confidence intervals for the outcomes of interest were not reported
Study design	Prospective observational studies (prospective cohort, case-cohort, or nested case-control)	Retrospective observational studies

### Data extraction and qualitative assessment

A standardized data-collection form was used to extract the following information from each included study: first author name, year of publication, cohort name, country of origin, follow-up duration, sex percentage, sample size, average age, type of DM, number of events, methods for dietary assessment, adjustments, and the maximally-adjusted risk estimates with 95% CIs.

The quality of the included studies was evaluated using the Newcastle-Ottawa Scale based on three domains: selection (maximum 4 points), comparability (maximum 2 points), and outcome (maximum 3 points) (39). The studies were then classified as high quality (NOS: ≥ 8–9), moderate quality (NOS: 6–7), or low quality (NOS: < 6). The certainty of the evidence was assessed using the NutriGrade scoring system (40), which offers a balanced evaluation of cohort studies in nutrition research through a continuous scoring approach. Unlike GRADE, which automatically rates observational studies as low certainty, NutriGrade allows a more nuanced assessment that reflects study quality and context better. This distinction is especially important in nutrition research, where prospective cohort studies often represent the only feasible source of long-term evidence for diet–disease relationships. The scoring system incorporates the following items: (1) risk of bias, study quality, and study limitations (maximum 2 points); (2) precision (maximum 1 point); (3) heterogeneity (maximum 1 point); (4) directness (maximum 1 point); (5) publication bias (maximum 1 point); (6) funding bias (maximum 1 point); (7) effect size (maximum 2 points); and (8) dose-response (maximum 1 point). The certainty of the evidence for each analysis was graded as very low (0 to < 4 points), low (4 to < 6 points), moderate (6 to < 8 points), or high (≥8 points).

### Statistical analysis

HR was chosen as the measure of association because it was the most common measure of association among the included studies. A random-effects model (41) was used to generate the summary HRs with their 95% CIs for the associations between tea or coffee consumption and the risks of CVD and all-cause and cause-specific mortality. In the main analysis, the risks of CVD and all-cause and cause-specific mortality associated with the highest vs. lowest category of tea or coffee consumption were estimated. However, these categories were defined differently across studies (e.g., daily, several times per week, or unspecified cut-offs), and the actual intake amounts in each category varied substantially. As such, comparisons between studies based on predefined intake frequencies may not be directly comparable or meaningful. To overcome this limitation and provide a more standardized risk estimate, we also analyzed tea or coffee consumption as a continuous variable, estimating the risk associated with each additional cup per day. This linear dose-response approach allows for more consistent interpretation across studies and reduces misclassification bias arising from heterogeneous category definitions. The method proposed by Greenland and Longnecker (42) and Orsini et al. (43) was used, which requires studies to report at least three categories of tea or coffee consumption and the distributions of events and person-years in each category. In addition, to assess whether the association was nonlinear, we applied restricted cubic spline regression models with knots at the 10th, 50th, and 90th percentiles of tea or coffee intake (44). The likelihood ratio test was used to determine the statistical significance of non-linearity (44). Subgroup and meta-regression analyses by gender, region, duration of follow-up, number of participants, and adjustment for potential confounders were performed to investigate potential sources of heterogeneity and effect modifiers. Heterogeneity between studies was assessed by the Q test and *I*^2^ statistics. *I*^2^ is the amount of total variation that is explained by between-study variation. *I*^2^ values of < 25%, 25–50%, and > 50% are considered low, moderate, and high heterogeneity, respectively. Publication bias was not assessed due to the low number of studies (n < 10) included in each analysis. We conducted sensitivity analyses that restricted the analyses to the studies that included only participants with T2DM. The potential publication bias was assessed using Begg's rank correlation test and Egger's linear regression (45). If the publication bias was detected, the trim and fill method was performed to adjust the bias (46). All statistical analyses were conducted by using Stata, version 16.0. All *P* values were two-sided, and the significance level was set at *P* < 0.05.

## Results

### Literature search and study characteristics

The flow chart of the literature search is shown in Supplementary Figure S1. Initial database searches retrieved 19,392 articles (2,951 from Pubmed, 8,464 from Web of Science, 417 from Cochrane Library and 10,511 from Embase). After removing duplicate articles and title/abstract screening, 196 articles were eligible for full-text review. Of these 196 articles, 56 were excluded because the studies investigated the association of interest in the general population and did not report risk estimates by DM status, and two papers were excluded because the study (35, 36) was performed in the same study populations as the included studies (24, 31) (Supplementary Appendix S1). Finally, 17 articles (18–34) were included in the present meta-analysis. These articles were published between 2003 and 2025. The characteristics of the included studies are summarized in Supplementary Table S2. Most included articles reported the findings mainly from American cohorts [the College Alumni Health Study, (18) the Nurses' Health Study (NHS) (20, 32), the Health Professionals Follow-up Study (HPFS) (21, 32), the National Institutes of Health-American Association of Retired Persons (NIH-AARP) Diet and Health Study (22), the Prostate, Lung, Colorectal, and Ovarian (PLCO)

Cancer Screening Trial (23), and the National Health and Nutrition Examination Survey (NHANES) (26)]. Few others reported the findings from European (the North Karelia Project (19), the Moli-sani Study, (30) the United Kingdom Biobank (UKB) (31, 33), and the Alpha Omega Cohort (25) and Asian {Japan Public Health Center (JPHC)-Based Prospective Study (24), the Fukuoka Diabetes Registry (27), the China-PAR project (28), the China Kadoorie Biobank (CKB) (29) cohorts, and the China Comprehensive Research on the Prevention and Control of Diabetes (35)} cohorts. Other than the NHS (20, 32), which enrolled women only, and the HPFS (21, 32), which enrolled men only, other cohorts enrolled both men and women (but sex-specific results were rarely reported). The Fukuoka Diabetes Registry (27) and Comprehensive Research on the Prevention and Control of Diabetes (34) were the cohorts comprised entirely of patients with DM. In other cohorts, risk estimation on patients with DM was performed by excluding individuals without DM from their analysis (19–21, 26, 29, 32, 33) or stratifying their analysis by DM status (18, 22–25, 28, 30, 31). Several studies (19–21, 27, 32–34) restricted the inclusion criteria to T2DM, while others (18, 22–26, 30, 31) included DM regardless of type. Coffee or tea consumption was evaluated using FFQ in all cohorts except the NHANES (26), which used 24-h dietary recall. Only the NHS (20, 32) and the HPFS (21, 32) performed multiple dietary assessments at baseline and follow-ups, whereas other studies relied on single-point assessments at baseline. In nearly all cohorts, study outcomes were ascertained by medical records, record linkage, or death certificates. All studies provided adjusted HRs. Based on the Newcastle–Ottawa scale score, 12 studies with a total score of ≥7 were classified as high quality (Supplementary Table S3).

### Meta-analyses

#### Coffee consumption

In the meta-analysis of the highest vs. lowest category of coffee consumption, the pooled HR (95%CI) was 0.82 (0.73, 0.91; Supplementary Figure S2A) for all-cause mortality (19, 22–27, 31, 32), 0.84 (0.67, 1.04; Supplementary Figure S3A) for CVD mortality (19, 25–27, 31, 32), 0.66 (0.51, 0.85; Supplementary Figure S4A) for CHD mortality (19, 20, 25), and 0.96 (0.62, 1.48; Supplementary Figure S5A) for cancer mortality (26, 27, 32), 0.85 (0.75, 0.97; Supplementary Figure S6A) for CVD incidence (32, 33), 0.82 (0.68, 0.99; Supplementary Figure S7A) for CHD incidence (20, 21, 33), and 0.95 (0.68, 1.34; Supplementary Figure S8A) for stroke incidence (20, 21, 33). High heterogeneity was observed in the analyses of all-cause mortality, CVD mortality, and cancer mortality (*I*^2^ ≥57.7%), whereas no heterogeneity was observed in the analyses of other outcomes (*I*^2^ = 0%). There was no evidence of publication bias with Begg's test (*P* ≥ 0.72) and Egger's test (*P* ≥ 0.29).

In the dose-response meta-analysis, the pooled HR (95%CI) for each-cup-per-day increase in coffee consumption was 0.96 (0.93, 0.98; Supplementary Figure S2B) for all-cause mortality (19, 22–27, 30, 32), 0.95 (0.94, 0.97; Supplementary Figure S3B) for CVD mortality (19, 25–27, 30, 32), 0.94 (0.91, 0.97; Supplementary Figure S4B) for CHD mortality (19, 20, 25), 0.96 (0.93, 1.00; Supplementary Figure S5B) for cancer mortality (26, 27, 32), 0.97 (0.95, 0.99; Supplementary Figure S6B) for CVD incidence (32, 33), 0.96 (0.93, 0.99; Supplementary Figure S7B) for CHD incidence (20, 21, 33), and 0.98 (0.93, 1.04; Supplementary Figure S8B) for stroke incidence (20, 21, 33). High heterogeneity was observed in the analysis of all-cause mortality (*I*^2^ = 77.1%), whereas no heterogeneity was observed in the analyses of other outcomes (*I*^2^ = 0%). There was no evidence of publication bias with Begg's test (*P* ≥ 0.11) and Egger's test (*P* ≥ 0.19).

**Figure 1:**
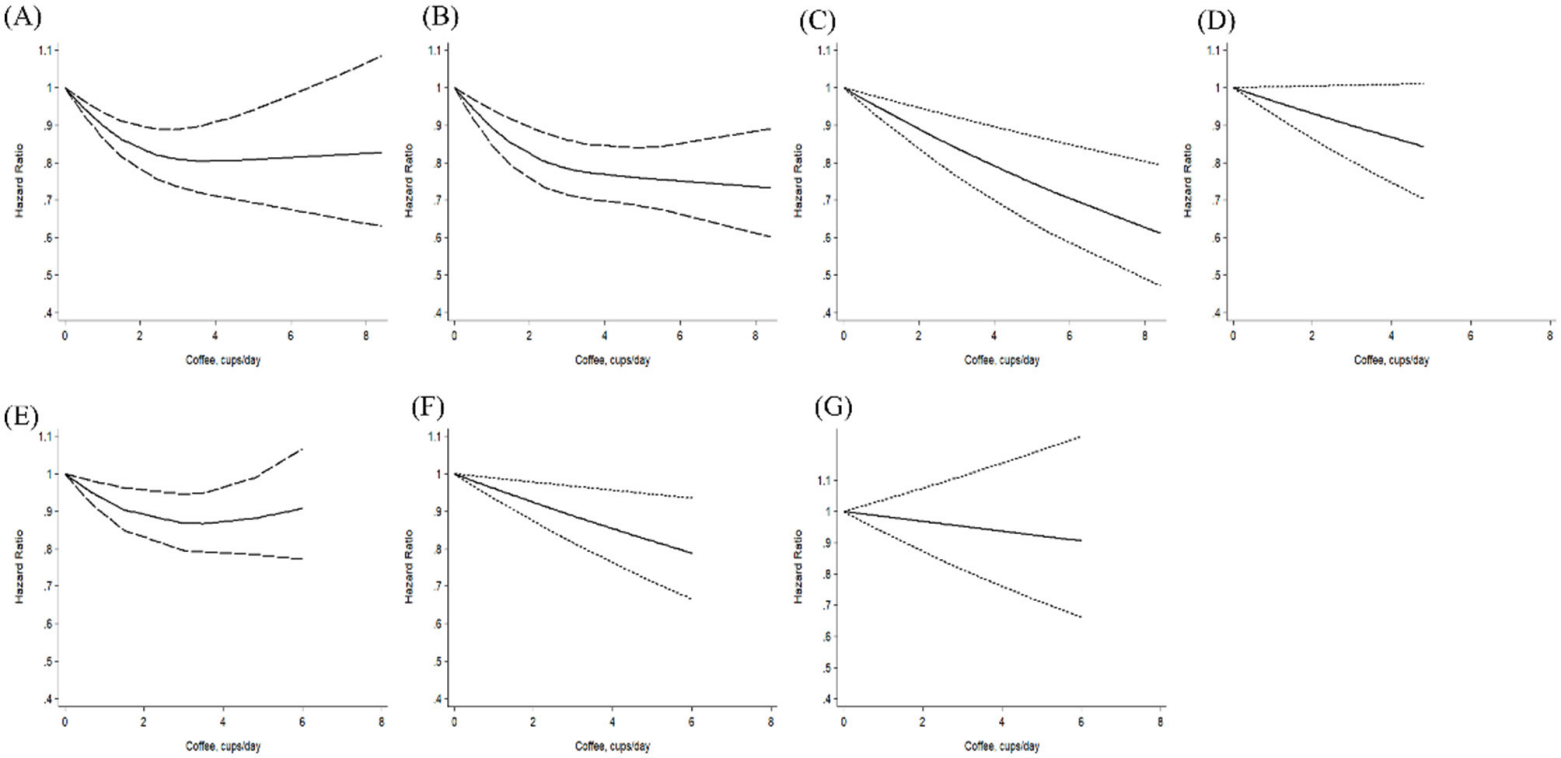
Nonlinear dose-response association of coffee consumption with the risks of **(A)** all-cause mortality (*P* nonlinearity < 0.01), **(B)** CVD mortality (*P* nonlinearity = 0.43), **(C)** CHD mortality (*P* nonlinearity = 0.79), **(D)** cancer mortality (*P* nonlinearity = 0.67), **(E)** CVD incidence (*P* nonlinearity =0.37), **(F)** CHD incidence (*P* nonlinearity = 0.25), and **(G)** stroke incidence (*P* nonlinearity = 0.82) in individuals with diabetes mellitus.

The association between coffee consumption and the risk of all-cause mortality was nonlinear (*P* non-linearity < 0.01), with a steep risk reduction observed at one to four cups per day, followed by a plateau (Figure 1A). The lowest risk of all-cause mortality was observed at approximately four cups per day. Although an inverse association persisted beyond four cups, the 95% CIs widened and became nonsignificant above six cups. No evidence of a nonlinear association was found for other outcomes (*P* non-linearity≥ 0.25; Figures 1B–G).

Although we included studies regardless of the type of DM, the analyses of CVD, CHD, and stroke incidence were completely comprised of studies that included only participants with T2DM. When the analyses of all-cause mortality, CVD mortality, CHD mortality, and cancer mortality were restricted to studies including only participants with T2DM, the results of the highest vs. lowest category and dose-response meta-analyses did not materially alter (Supplementary Figure S9).

Subgroup and meta-regression analyses were performed only for the association between coffee consumption and the risk of all-cause mortality, as there were not enough studies for meaningful analyses of other associations. In both highest vs. lowest and linear dose-response analyses, a tendency toward a lower risk of all-cause mortality was observed across subgroups, although it did not always reach statistical significance (Table 2). The inverse association appeared stronger in studies investigating T2DM compared to those including any type of DM (*P* meta-regression = 0.008; highest vs. lowest only) and studies that adjusted for diabetes duration and glycemic control (*P* meta-regression ≥ 0.018; highest vs. lowest and linear dose-response). The high heterogeneity observed in the main analysis disappeared or attenuated in certain subgroups, particularly in the highest vs. lowest analyses among men, American studies, studies on T2DM, studies that did not adjust for physical activity, and studies that adjusted for diabetes duration and glycemic control. Similarly, in linear dose-response analyses, heterogeneity was reduced in studies among men, those adjusting for hypoglycemic drugs, and those that did not adjust for smoking status or BMI, drinking, or smoking.

**Table 2:** Subgroup and meta-regression analyses of the association between coffee consumption and the risk of all-cause mortality in individuals with diabetes mellitus.

**Subgroup**	**Highest vs. lowest category**					**Dose-response (per 1 cup/day increase)**				
	***N***	**References**	**HR (95% CI)**	*I*^2^ **(%)**	***P*** **meta-regression**	***N***	**References**	**HR (95% CI)**	*I*^2^ **(%)**	***P*** **meta-regression**
All studies	9	(19, 22–27, 31, 32)	**0.82 (0.73, 0.91)**	55.7		9	(19–27, 30, 32)	**0.96 (0.93, 0.98)**	77.1	
**Gender**					0.995					0.585
Men	4	(21, 22, 26, 24)	**0.85 (0.76, 0.94)**	0.0		4	(21, 22, 24, 26)	**0.95 (0.93, 0.97)**	0.0	
Women	4	(20, 22, 26, 24)	**0.78 (0.62, 0.97)**	39.3		4	(20, 22, 24, 26)	0.90 (0.81, 1.00)	51.6	
All	6	(19, 23, 25, 27, 31, 32)	**0.80 (0.67, 0.95)**	78.7		6	(19, 23, 25, 27, 30, 32)	**0.95 (0.92, 0.99)**	83.7	
**Geographical region**					0.856					0.752
Europe	3	(19, 25, 31)	0.91 (0.69, 1.20)	83.3		3	(19, 25, 30)	0.99 (0.93, 1.05)	59.5	
Asia	2	(23, 27)	0.75 (0.46, 1.22)	81.0		2	(24, 27)	0.89 (0.74, 1.07)	84.3	
USA	4	(22, 24, 26, 32)	**0.80(0.74, 0.86)**	0.0		4	(22, 23, 26, 32)	**0.95(0.92, 0.98)**	83.6	
**Number of participants**					0.663					0.490
< 5,000	6	(19, 23–27)	**0.80 (0.67, 0.95)**	60.2		6	(19, 23–27)	0.97 (0.93, 1.00)	66.5	
>5,000	3	(22, 31, 32)	**0.85 (0.73, 0.98)**	78.4		3	(22.30, 32)	**0.94 (0.91, 0.98)**	79.4	
**Follow-up duration**					0.778					0.651
< 10 years	4	(24–27)	0.80 (0.62, 1.04)	66.9		5	(23, 25–27, 30)	0.95 (0.88, 1.03)	69.5	
>10 years	5	(19, 22, 23, 31, 32)	**0.83 (0.73, 0.94)**	72.5		4	(19, 22, 24, 32)	**0.95 (0.93, 0.97)**	73.6	
**Type of DM**					0.030					0.128
Any DM	6	(22–26, 31)	**0.89 (0.80, 0.99)**	50.7		6	(22–26, 30)	**0.97 (0.95, 0.99)**	32.2	
T2DM	3	(19, 27, 32)	**0.70 (0.63, 0.79)**	0		3	(19, 27, 32)	**0.92 (0.88, 0.97)**	85.1	
**Adjustments**										
**BMI, alcohol consumption, and smoking**					-					0.902
Yes	8	(22–27, 31, 32)	**0.84 (0.75, 0.94)**	65.5		7	(22, 24, 23, 25, 26, 27, 32)	**0.96 (0.92, 0.99)**	80.9	
No	1	(19)	**0.70 (0.58, 0.84)**	-		2	(19, 30)	**0.96 (0.94, 0.98)**	0	
**Hypoglycemic drugs**					0.577					0.055
Yes	2	(26, 32)	**0.75 (0.66, 0.86)**	0.0		2	(26, 32)	**0.92 (0.90, 0.94)**	0.0	
No	7	(19, 22–25, 27, 31)	**0.84 (0.73, 0.95)**	71.9		7	(19.22–27, 30)	**0.97 (0.95, 0.99)**	61.0	
**Energy intake**					0.834					-
Yes	7	(22–27, 32)	**0.81 (0.72, 0.91)**	50.5		8	(22–27, 30, 32)	**0.96 (0.93, 0.99)**	77.7	
No	2	(19, 31)	0.83 (0.60, 1.14)	89.2		1	(19)	**0.96 (0.94, 0.98)**	-	
**Smoking status**					0.848					0.797
Yes	7	(22, 23, 25, 26, 27, 31, 32)	**0.83 (0.73, 0.94)**	69.5		7	(22, 23, 25–27, 30, 32)	**0.95 (0.92, 0.99)**	80.8	
No	2	(19, 24)	0.80 (0.60, 1.08)	71.1		2	(19, 24)	**0.96 (0.94, 0.98)**	0	
**Physical activity**					0.072					0.520
Yes	5	(22, 24, 25, 31, 32)	0.89 (0.78, 1.01)	69.5		5	(22, 24, 25, 30, 32)	0.96 (0.93, 1.00)	76.5	
No	4	(19, 23, 26, 27)	**0.72 (0.63, 0.81)**	0.0		4	(19, 23, 26, 27)	**0.95 (0.92, 0.99)**	71.9	
**Diabetes duration and glycemic control**					0.164					0.018
Yes	3	(26, 27, 32)	**0.72 (0.63, 0.83)**	14.7		3	(26, 27, 32)	**0.88 (0.80, 0.97)**	53.6	
No	6	(19, 22, 24, 23, 25, 31)	**0.87 (0.76, 0.98)**	68.1		6	(19, 22–25, 30)	**0.97 (0.95, 0.98)**	32.6	

Bold value indicates *P* < 0.05.

#### Tea consumption

In the meta-analysis of the highest vs. lowest category of tea consumption, the pooled HR (95%CI) was 0.85 (0.79, 0.92; Supplementary Figure S10A) for all-cause mortality (27–29, 31, 32, 34), 0.86 (0.80, 0.93; Supplementary Figure S11A) for CVD mortality (27–29, 31, 32, 34), 0.95 (0.76, 1.19; Supplementary Figure S12A) for cancer mortality (27, 32, 34), and 0.96 (0.84, 1.10; Supplementary Figure S13) for CVD incidence (18, 32). High heterogeneity was observed for all-cause mortality (*I*^2^ ≥ 46.2%), whereas no heterogeneity was found for other outcomes (*I*^2^ = 0%). There was no evidence of publication bias based on Begg's test (*P* ≥ 0.13) and Egger's test (*P* ≥ 0.14).

In the dose-response meta-analysis, the pooled HR and 95%CI for each-cup-per-day increase in tea consumption was 0.93 (0.88, 0.96; Supplementary Figure S10B) for all-cause mortality (27, 29, 32, 34), 0.92 (0.88, 0.96; Supplementary Figure S11B) for CVD mortality (27, 32, 34), and 0.99 (0.93, 1.05; Supplementary Figure S12B) for cancer mortality (27, 32, 34). High heterogeneity was observed for all-cause mortality (*I*^2^ = 64.8%), whereas no heterogeneity was found for other outcomes (*I*^2^ = 0%). There was no evidence of publication bias based on Begg's test (*P* ≥ 0.73) and Egger's test (*P* ≥ 0.12).

**Figure 2:**
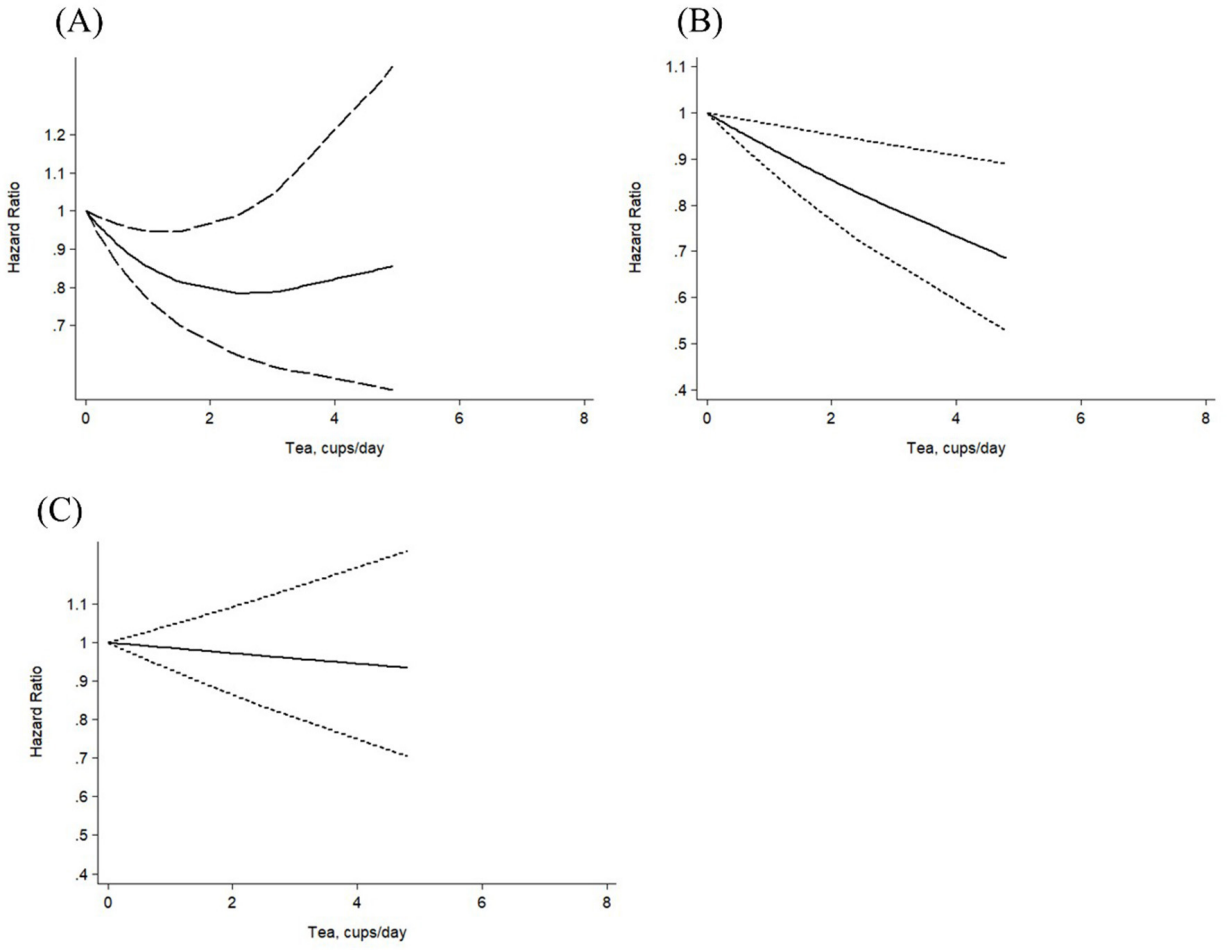
Nonlinear Dose-response association of tea consumption with the risks of **(A)** all-cause mortality (*P* nonlinearity < 0.01), **(B)** CVD mortality (*P* nonlinearity = 0.26), and **(C)** cancer mortality (*P* nonlinearity = 0.66) in individuals with diabetes mellitus.

The association between tea consumption and the risk of all-cause mortality was nonlinear (*P* non-linearity < 0.01), with the greatest risk reduction observed at up to 2 cups per day (Figure 2A). Although an inverse association persisted beyond 2 cups, the 95% CI became wider and lost statistical significance. No evidence of nonlinearity was observed for other outcomes (*P* non-linearity ≥0.26; Figures 2B, C).

Only analysis of cancer mortality was fully comprised of studies that included only participants with T2DM. When restricting the analyses of all-cause mortality and CVD mortality to studies including only participants with T2DM (Supplementary Figure S14), the inverse association remained significant for all-cause mortality in the highest vs. lowest and linear dose-response analyses. The inverse association for CVD mortality became statistically insignificant in the highest vs. lowest analysis but remained in the dose-response analysis. Restriction to T2DM could not be applied to the analysis of CVD incidence due to the limited studies (*n* = 2; 1 T2DM and 1 any DM).

### The certainty of the evidence

For coffee consumption, the certainty of the evidence was “low” for cancer mortality and stroke incidence and “moderate” for all-cause mortality, CVD mortality, CHD mortality, CVD incidence, and CHD incidence (Supplementary Table S4). For tea consumption, the NutriGrade meta-evidence rating was “low” for all-cause mortality, cancer mortality, and CVD incidence and “moderate” for CVD mortality (Supplementary Table S4).

## Discussion

The present meta-analysis of published prospective observational studies provides up-to-date quantitative evidence on the associations of two of tea and coffee consumption with the risks of CVD and all-cause and cause-specific mortality in individuals with DM who are at increased risks of developing CVD and premature death. Compared with the lowest consumption category, the highest coffee consumption was associated with lower risks of all-cause mortality, CVD mortality, CHD mortality, CVD incidence, and CHD incidence. Similarly, the highest tea consumption was associated with lower risks of all-cause and CVD mortality. Each additional daily cup was linearly associated with a 5% lower risk of CVD mortality, 6% lower risk of CHD mortality, 3% lower risk of CVD incidence, and 4% lower risk of CHD incidence for coffee, and an 8% lower risk of CVD mortality for tea. Coffee or tea consumption was non-linearly associated with the risk of all-cause mortality, with the greatest risk reduction at one to four cups of coffee per day or up to two cups of tea per day. Although all included studies were classified as high quality based on the Newcastle–Ottawa Scale, the certainty of the evidence, as assessed by the NutriGrade system, was mostly graded as moderate for coffee consumption (except for cancer mortality and stroke incidence, which were graded as low) and low for tea consumption (except for CVD mortality, which was graded as moderate). This discrepancy primarily reflects imprecision in effect estimates, limited evidence for dose–response relationships, small effect sizes, and potential publication bias. These limitations reduce confidence in the observed associations and underscore the need for further high-quality research to strengthen the evidence base. While the associations between tea consumption and the risks of CVD and all-cause and cause-specific mortality in individuals with DM have not been previously quantified, the associations between coffee consumption and the risks of CVD and all-cause and cause-specific mortality in individuals with DM have been quantitatively assessed in a meta-analysis (37). However, this meta-analysis did not include one (30) missing article that was available at that time and four (31–34) recently published articles, three of which reported the updated findings from several studies (20, 21, 35). These studies were all included in the present meta-analysis. Moreover, the previous meta-analysis defined its study population as exclusively T2DM but included studies that did not distinguish DM type, leading to potential misinterpretation. In contrast, our meta-analysis included all DM types and found that restricting the analysis to T2DM-specific studies did not substantially alter results. Both analyses found inverse dose-response associations between coffee consumption and all-cause, CVD, and CHD mortality, with no association with cancer mortality. However, only the present analysis identified significant associations with CVD and CHD incidence, likely due to the inclusion of updated studies (31–34).

Individuals with DM are more likely to develop CVD and die prematurely from all or specific causes than those without this condition (1–4). Tea (13–15) and coffee (16, 17) consumption have been linked to lower risks of several chronic diseases, including CVD, and death from all and specific causes and in general populations. Our findings suggest that the previously observed associations of coffee and tea consumption with potential cardiometabolic and longevity benefits in general populations may also be present in individuals with DM, offering preliminary evidence that may inform future research and discussions around dietary guidance for DM populations. While the present findings are somewhat encouraging in the way that drinking low-cost and easily accessible coffee and tea regularly as a part of a healthy diet might be associated with a lower risk of CVD and premature death in at-risk DM populations, the certainty of the evidence on all investigated associations was deemed moderate or low. Therefore, these findings should be interpreted with caution. Nevertheless, in the context of a balanced and health-promoting dietary pattern, moderate consumption of coffee or tea may represent a potentially favorable dietary behavior for individuals with DM, although further research is needed. Healthcare professionals might take these associations into account when discussing dietary patterns with patients, while emphasizing the preliminary nature of the evidence. Further effort should be made to explore the lesser-known aspects of the associations in DM, such as the potential effect modification by sugar content (i.e., unsweetened and sweetened), types of tea (i.e., black and green tea), and caffeine content (i.e., caffeinated and decaffeinated).

The biological mechanisms underlying the inverse associations between coffee or green tea consumption and some investigated outcomes in individuals with DM are not fully understood. However, several pathways have been proposed, particularly those related to oxidative stress, inflammation, and metabolic regulation. In DM, chronic hyperglycemia can lead to excessive production of reactive oxygen species and reactive nitrogen species, which damage cellular components such as DNA, proteins, and lipids. This oxidative stress contributes to β-cell dysfunction and impaired insulin secretion, which are central to the development and progression of T2DM and its complications, including increased risks of cardiovascular disease and premature mortality (47).

Coffee and tea are major sources of dietary polyphenols in many populations (48–50). Although both beverages are rich in polyphenols, their specific compositions differ: coffee primarily contains chlorogenic acids, a subclass of hydroxycinnamic acids, while green tea is particularly rich in catechins, which belong to the flavanol class (51, 52). These polyphenolic compounds possess potent antioxidant properties, including the ability to scavenge free radicals and regulate redox-sensitive signaling pathways, thereby reducing oxidative damage. A key mechanism involves the activation of nuclear factor erythroid 2–related factor 2 (Nrf2), a transcription factor that upregulates genes responsible for antioxidant defense, mitochondrial biogenesis, and cellular protection (53). Through this mechanism, polyphenols may preserve mitochondrial function and limit β-cell damage during periods of high insulin demand, such as in prediabetes and early DM.

Furthermore, polyphenols exert notable anti-inflammatory effects. They can suppress the expression of proinflammatory cytokines such as interferon-γ, tumor necrosis factor-α, and various chemokines in different cell types (54), potentially mitigating the chronic low-grade inflammation commonly observed in individuals with T2DM and CVD. Higher intake of polyphenols from coffee or tea has also been associated with a lower risk of mortality, particularly from CVD (52).

Some mechanisms may be more specific to coffee. For instance, chlorogenic acids have demonstrated protective effects on pancreatic β-cells under oxidative stress and support glucose homeostasis. Coffee consumption has also been associated with altered secretion of gastrointestinal hormones, including increased gastric inhibitory polypeptide and decreased glucagon-like peptide-1, which may affect postprandial glucose absorption and insulin sensitivity (55, 56).

Alternatively, coffee or tea may displace sugar-sweetened beverages, which have been consistently linked to adverse metabolic and cardiovascular outcomes (32). While this displacement effect is not unique to coffee or tea (i.e., other non-caloric beverages such as water may yield similar benefits) coffee and tea may provide additional advantages due to their bioactive compound profiles. Thus, replacing sugar-sweetened beverages with coffee or tea may not only reduce harmful added sugar intake but also introduce bioactive constituents that support cardiometabolic health.

Glycemic control in individuals with DM is closely linked to lifestyle factors (57) and is a key determinant of CVD and mortality risk (58). Healthier lifestyle choices, including adherence to a balanced diet, weight management, regular physical activity, non-smoking, and alcohol avoidance, may improve glycemic control, leading to lower risks of CVD and mortality. In the present meta-analysis, the inverse associations between coffee consumption and the risks of all-cause and CVD mortality remained significant in studies that adjusted for glucose control, diabetes duration, smoking, alcohol consumption, physical activity, or BMI. However, for other associations, limited data prevented further assessment. The impact of dietary factors remains unclear, as the included studies varied in their approach to dietary adjustments, with most focusing on nutrient intake or specific food groups rather than overall dietary patterns. Notably, the inverse associations between coffee consumption and the risks of all-cause and CVD mortality persisted in studies that accounted for energy intake. Future research should incorporate comprehensive dietary assessments to better address potential confounding.

Several caveats need to be considered while interpreting the results of the present meta-analysis. Firstly, the observational design of the included studies introduces an inherent risk of residual or unmeasured confounding. Although most studies adjusted for key covariates such as age, sex, BMI, smoking, alcohol use, and physical activity, other potentially influential factors—such as diabetes duration, glycemic control, medication use, comorbid conditions, and underlying dietary pattern—were not consistently accounted for. These uncontrolled variables may partially account for the observed associations. Future research should incorporate more comprehensive confounder adjustment or alternative approaches such as Mendelian randomization to improve causal inference. Secondly, the majority of included studies relied on single-time, self-reported dietary assessments, which are susceptible to measurement error and may not reflect long-term intake, especially if dietary habits change over time. This limitation can result in exposure misclassification and imprecise estimation of associations. Repeated assessments or the use of dietary biomarkers may help improve the accuracy of exposure measurement. Thirdly, heterogeneity across studies on certain outcomes is an important limitation. Although subgroup and meta-regression analyses were conducted for the association between coffee consumption and the risk of all-cause mortality, some heterogeneity remained unexplained, potentially reflecting differences in uncaptured population and study characteristics. This may reduce the precision of the pooled estimates and complicate their interpretation. For other associations, the limited number of studies precluded formal exploration of heterogeneity, which restricts the ability to assess consistency across different population and study characteristics. Finally, although publication bias was not evident in all analyses, the assessment of publication bias is often underpowered, particularly when fewer than 10 studies were available. Therefore, the possibility of selective publication of studies with positive results cannot be ruled out. In this case, small studies with null or negative findings may remain unpublished, which could overestimate the observed associations. Future meta-analyses with a greater pool of studies may allow for a more robust assessment of publication bias. Despite all the limitations above, the present meta-analysis provided the most comprehensive and up-to-date evidence on the association between tea or coffee consumption with the risks of CVD and all-cause and cause-specific mortality in individuals with DM and the first to quantify the association between tea or coffee consumption with the risks of CVD and all-cause and cause-specific mortality in this population.

## Conclusions

Accumulating evidence from prospective observational studies suggests that daily coffee or tea consumption may be associated with lower risks of CVD and death, particularly from CVD, among individuals with DM. However, given the observational nature of the studies, methodological limitations, and the low to moderate certainty of the evidence, these findings should be interpreted with caution. High-quality research is needed to further confirm these associations across diverse DM populations.

## Data Availability

The original contributions presented in the study are included in the article/Supplementary material; further inquiries can be directed to the corresponding author/s.
