# Supplementary material for: Coffee and tea consumption and cardiovascular disease and all-cause and cause-specific mortality in individuals with diabetes mellitus: a meta-analysis of prospective observational studies
Source: Front Nutr. 2025 Jun 2;12:1570644. doi: 10.3389/fnut.2025.1570644 (PMC12168468; doi:10.3389/fnut.2025.1570644)

**Table S1** Full detail of the search strategy^1^

| **Database** | **Step** | **Search Syntax** | **Total retrieves** |
| --- | --- | --- | --- |
| PubMed | 1 | Search (coffee OR caffeine OR beverage OR beverages OR tea OR "black tea" OR "green tea") | 281,567 |
|  | 2 | Search (mortality OR death OR survival OR "cardiovascular disease" OR "cardiovascular events" OR "coronary heart disease" OR "ischemic heart disease" OR "coronary events" OR "myocardial infarction" OR stroke OR CVD OR CHD OR IHD) | 4,388,935 |
|  | 3 | Search (longitudinal OR cohort OR “nested case-control” OR "follow-up" OR prospective OR prospectively OR observational) | 3,733,120 |
|  | 4 | Search (#1 AND #2 AND #3) | 2,951 |
| Web of Science | 1 | (coffee OR caffeine OR beverage OR beverages OR tea OR "black tea" OR "green tea") AND (mortality OR death OR survival OR "cardiovascular disease" OR "cardiovascular events" OR "coronary heart disease" OR "ischemic heart disease" OR "coronary events" OR "myocardial infarction" OR stroke OR CVD OR CHD OR IHD) AND (longitudinal OR cohort OR “nested case-control” OR "follow-up" OR prospective OR prospectively OR observational) | 8,464 |
| Cochrane Library | 1 | MeSH descriptor: [Coffee] explode all trees OR (coffee):ti,ab,kw OR (caffeine):ti,ab,kw OR (beverage):ti,ab,kw OR (beverages):ti,ab,kw OR (tea):ti,ab,kw OR (black tea):ti,ab,kw OR (green tea):ti,ab,kw | 18,727 |
|  | 2 | MeSH descriptor: [mortality] explode all trees OR (mortality):ti,ab,kw OR (death):ti,ab,kw OR (survival):ti,ab,kw OR (cardiovascular disease):ti,ab,kw OR (cardiovascular events):ti,ab,kw OR (coronary heart disease):ti,ab,kw OR (ischemic heart disease):ti,ab,kw OR (coronary events):ti,ab,kw OR (myocardial infarction):ti,ab,kw OR (stroke):ti,ab,kw OR (CVD):ti,ab,kw OR (CHD):ti,ab,kw OR (IHD):ti,ab,kw | 33,298 |
|  | 3 | (longitudinal):ti,ab,kw OR (cohort):ti,ab,kw OR (nested case-control):ti,ab,kw OR (follow-up):ti,ab,kw OR (prospective):ti,ab,kw OR (prospectively):ti,ab,kw OR (observational):ti,ab,kw | 609,518 |
|  | 4 | #1 AND #2 AND #3 | 417 |
| Embase | 1 | ‘coffee’ OR ‘caffeine’ OR ‘beverage’ OR ‘beverages’ OR ‘tea’ OR ‘black tea’ OR ‘green tea’ | 439,331 |
|  | 2 | ‘mortality’ OR ‘death’ OR ‘survival’ OR ‘cardiovascular disease’ OR ‘cardiovascular events’ OR ‘coronary heart disease’ OR ‘ischemic heart disease’ OR ‘coronary events’ OR ‘myocardial infarction’ OR ‘stroke’ OR ‘CVD’ OR ‘CHD’ OR ‘IHD’ | 10,096,573 |
|  | 3 | ‘longitudinal’ OR ‘cohort’ OR ‘nested case-control’ OR ‘follow-up’ OR ‘prospective’ OR ‘prospectively’ OR ‘observational’ | 5,877,888 |
|  | 4 | #1 AND #2 AND #3 | 10,511 |

^1^All databases were last searched on April 23rd, 2025.

**Figure S1** Flow chart of the study selection process


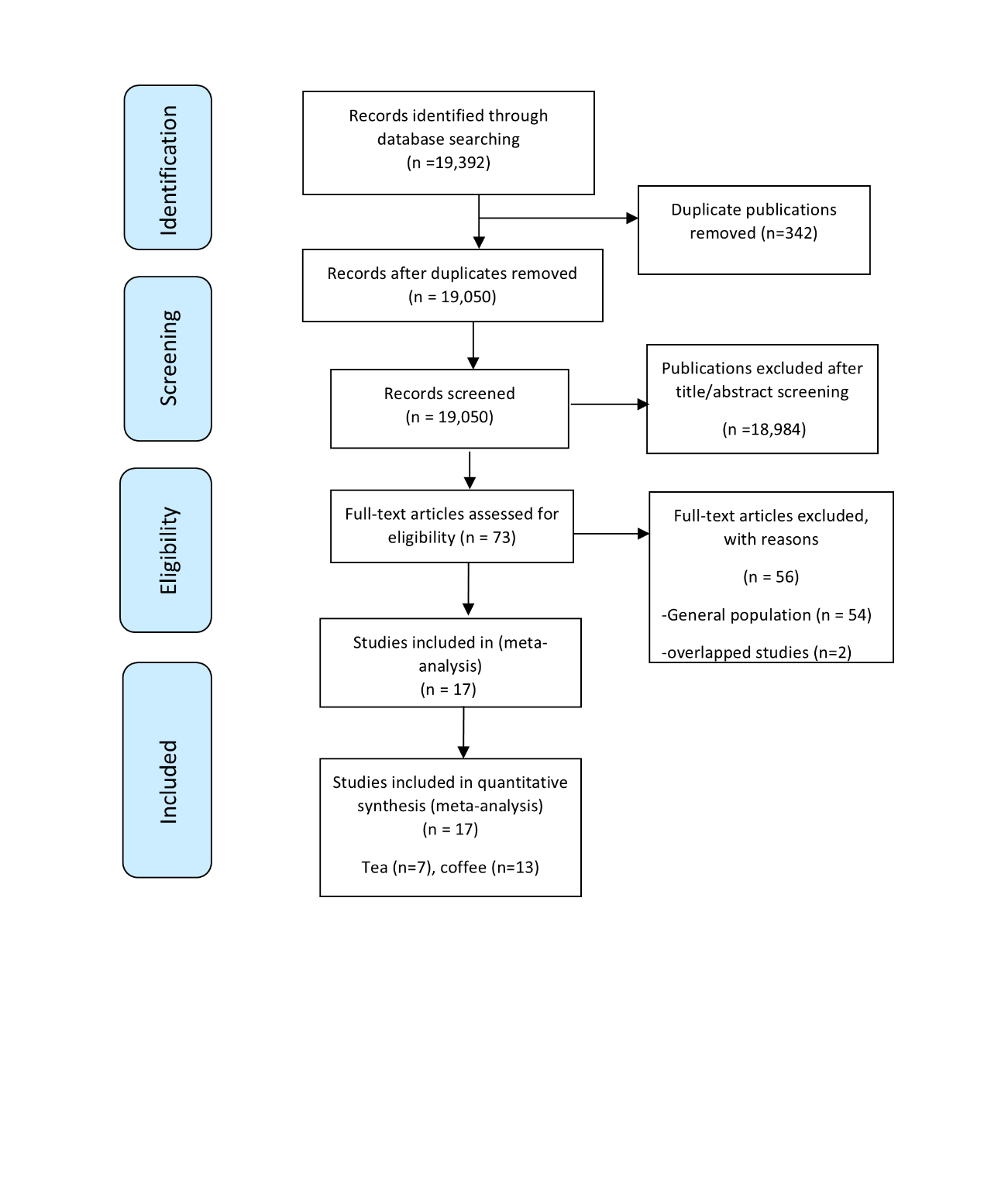


**Supplementary appendix**

**References of the excluded studies**

*The studies investigated the general population and did not perform stratification by diabetes status*

1. Jacobsen BK, Bjelke E, Kvåle G, Heuch I. Coffee drinking, mortality, and cancer incidence: results from a Norwegian prospective study. J Natl Cancer Inst. 1986 May;76(5):823-31.
2. Vandenbroucke JP, Kok FJ, van 't Bosch G, van den Dungen PJ, van der Heide-Wessel C, van der Heide RM. Coffee drinking and mortality in a 25-year follow up. Am J Epidemiol. 1986 Feb;123(2):359-61.
3. LeGrady D, Dyer AR, Shekelle RB, Stamler J, Liu K, Paul O, Lepper M, Shryock AM. Coffee consumption and mortality in the Chicago Western Electric Company Study. Am J Epidemiol. 1987 Nov;126(5):803-12.
4. Rosengren A, Wilhelmsen L. Coffee, coronary heart disease and mortality in middle-aged Swedish men: findings from the Primary Prevention Study. J Intern Med. 1991 Jul;230(1):67-71.
5. Klatsky AL, Friedman GD, Armstrong MA. Coffee use prior to myocardial infarction restudied: heavier intake may increase the risk. Am J Epidemiol. 1990 Sep;132(3):479-88.
6. Hart C, Smith GD. Coffee consumption and coronary heart disease mortality in Scottish men: a 21 year follow up study. J Epidemiol Community Health. 1997 Aug;51(4):461-2.
7. Kleemola P, Jousilahti P, Pietinen P, Vartiainen E, Tuomilehto J. Coffee consumption and the risk of coronary heart disease and death. Arch Intern Med. 2000 Dec 11-25;160(22):3393-400.
8. Nakachi K, Matsuyama S, Miyake S, Suganuma M, Imai K. Preventive effects of drinking green tea on cancer and cardiovascular disease: epidemiological evidence for multiple targeting prevention. Biofactors. 2000;13(1-4):49-54.
9. Geleijnse JM, Launer LJ, Van der Kuip DA, Hofman A, Witteman JC. Inverse association of tea and flavonoid intakes with incident myocardial infarction: the Rotterdam Study. Am J Clin Nutr. 2002 May;75(5):880-6.
10. Jazbec A, Simić D, Corović N, Duraković Z, Pavlović M. Impact of coffee and other selected factors on general mortality and mortality due to cardiovascular disease in Croatia. J Health Popul Nutr. 2003 Dec;21(4):332-40.
11. Andersen LF, Jacobs DR Jr, Carlsen MH, Blomhoff R. Consumption of coffee is associated with reduced risk of death attributed to inflammatory and cardiovascular diseases in the Iowa Women's Health Study. Am J Clin Nutr. 2006 May;83(5):1039-46.
12. Kuriyama S, Shimazu T, Ohmori K, Kikuchi N, Nakaya N, Nishino Y, Tsubono Y, Tsuji I. Green tea consumption and mortality due to cardiovascular disease, cancer, and all causes in Japan: the Ohsaki study. JAMA. 2006 Sep 13;296(10):1255-65.
13. Happonen P, Läärä E, Hiltunen L, Luukinen H. Coffee consumption and mortality in a 14-year follow-up of an elderly northern Finnish population. Br J Nutr. 2008 Jun;99(6):1354-61.
14. Tanabe N, Suzuki H, Aizawa Y, Seki N. Consumption of green and roasted teas and the risk of stroke incidence: results from the Tokamachi-Nakasato cohort study in Japan. Int J Epidemiol. 2008 Oct;37(5):1030-40.
15. Suzuki E, Yorifuji T, Takao S, Komatsu H, Sugiyama M, Ohta T, Ishikawa-Takata K, Doi H. Green tea consumption and mortality among Japanese elderly people: the prospective Shizuoka elderly cohort. Ann Epidemiol. 2009 Oct;19(10):732-9.
16. de Koning Gans JM, Uiterwaal CS, van der Schouw YT, Boer JM, Grobbee DE, Verschuren WM, Beulens JW. Tea and coffee consumption and cardiovascular morbidity and mortality. Arterioscler Thromb Vasc Biol. 2010 Aug;30(8):1665-71.
17. Leurs LJ, Schouten LJ, Goldbohm RA, van den Brandt PA. Total fluid and specific beverage intake and mortality due to IHD and stroke in the Netherlands Cohort Study. Br J Nutr. 2010 Oct;104(8):1212-21.
18. Sugiyama K, Kuriyama S, Akhter M, Kakizaki M, Nakaya N, Ohmori-Matsuda K, Shimazu T, Nagai M, Sugawara Y, Hozawa A, Fukao A, Tsuji I. Coffee consumption and mortality due to all causes, cardiovascular disease, and cancer in Japanese women. J Nutr. 2010 May;140(5):1007-13.
19. Mineharu Y, Koizumi A, Wada Y, Iso H, Watanabe Y, Date C, Yamamoto A, Kikuchi S, Inaba Y, Toyoshima H, Kondo T, Tamakoshi A; JACC study Group. Coffee, green tea, black tea and oolong tea consumption and risk of mortality from cardiovascular disease in Japanese men and women. J Epidemiol Community Health. 2011 Mar;65(3):230-40.
20. Tamakoshi A, Lin Y, Kawado M, Yagyu K, Kikuchi S, Iso H; JACC Study Group. Effect of coffee consumption on all-cause and total cancer mortality: findings from the JACC study. Eur J Epidemiol. 2011 Apr;26(4):285-93.
21. Gardener H, Rundek T, Wright CB, Elkind MS, Sacco RL. Coffee and tea consumption are inversely associated with mortality in a multiethnic urban population. J Nutr. 2013 Aug;143(8):1299-308.
22. Kokubo Y, Iso H, Saito I, Yamagishi K, Yatsuya H, Ishihara J, Inoue M, Tsugane S. The impact of green tea and coffee consumption on the reduced risk of stroke incidence in Japanese population: the Japan public health center-based study cohort. Stroke. 2013 May;44(5):1369-74.
23. Liu J, Sui X, Lavie CJ, Hebert JR, Earnest CP, Zhang J, Blair SN. Association of coffee consumption with all-cause and cardiovascular disease mortality. Mayo Clin Proc. 2013 Oct;88(10):1066-74.
24. Löf M, Sandin S, Yin L, Adami HO, Weiderpass E. Prospective study of coffee consumption and all-cause, cancer, and cardiovascular mortality in Swedish women. Eur J Epidemiol. 2015 Sep;30(9):1027-34.
25. Odegaard AO, Koh WP, Yuan JM, Pereira MA. Beverage habits and mortality in Chinese adults. J Nutr. 2015 Mar;145(3):595-604.
26. Grosso G, Stepaniak U, Micek A, Stefler D, Bobak M, Pajak A. Coffee consumption and mortality in three Eastern European countries: results from the HAPIEE (Health, Alcohol and Psychosocial factors In Eastern Europe) study. Public Health Nutr. 2017 Jan;20(1):82-91.
27. Loomba RS, Aggarwal S, Arora RR. The Effect of Coffee and Quantity of Consumption on Specific Cardiovascular and All-Cause Mortality: Coffee Consumption Does Not Affect Mortality. Am J Ther. 2016 Jan-Feb;23(1):e232-7.
28. Nordestgaard AT, Nordestgaard BG. Coffee intake, cardiovascular disease and all-cause mortality: observational and Mendelian randomization analyses in 95 000-223 000 individuals. Int J Epidemiol. 2016 Dec 1;45(6):1938-1952.
29. Gapstur SM, Anderson RL, Campbell PT, Jacobs EJ, Hartman TJ, Hildebrand JS, Wang Y, McCullough ML. Associations of Coffee Drinking and Cancer Mortality in the Cancer Prevention Study-II. Cancer Epidemiol Biomarkers Prev. 2017 Oct;26(10):1477-1486.
30. Gunter MJ, Murphy N, Cross AJ, Dossus L, Dartois L, Fagherazzi G, Kaaks R, Kühn T, Boeing H, Aleksandrova K, Tjønneland A, Olsen A, Overvad K, Larsen SC, Redondo Cornejo ML, Agudo A, Sánchez Pérez MJ, Altzibar JM, Navarro C, Ardanaz E, Khaw KT, Butterworth A, Bradbury KE, Trichopoulou A, Lagiou P, Trichopoulos D, Palli D, Grioni S, Vineis P, Panico S, Tumino R, Bueno-de-Mesquita B, Siersema P, Leenders M, Beulens JWJ, Uiterwaal CU, Wallström P, Nilsson LM, Landberg R, Weiderpass E, Skeie G, Braaten T, Brennan P, Licaj I, Muller DC, Sinha R, Wareham N, Riboli E. Coffee Drinking and Mortality in 10 European Countries: A Multinational Cohort Study. Ann Intern Med. 2017 Aug 15;167(4):236-247.
31. Lim WH, Wong G, Lewis JR, Lok CE, Polkinghorne KR, Hodgson J, Lim EM, Prince RL. Total volume and composition of fluid intake and mortality in older women: a cohort study. BMJ Open. 2017 Mar 24;7(3):e011720.
32. Miller PE, Zhao D, Frazier-Wood AC, Michos ED, Averill M, Sandfort V, Burke GL, Polak JF, Lima JAC, Post WS, Blumenthal RS, Guallar E, Martin SS. Associations of Coffee, Tea, and Caffeine Intake with Coronary Artery Calcification and Cardiovascular Events. Am J Med. 2017 Feb;130(2):188-197.e5.
33. Park SY, Freedman ND, Haiman CA, Le Marchand L, Wilkens LR, Setiawan VW. Association of Coffee Consumption With Total and Cause-Specific Mortality Among Nonwhite Populations. Ann Intern Med. 2017 Aug 15;167(4):228-235.
34. Park SY, Freedman ND, Haiman CA, Le Marchand L, Wilkens LR, Setiawan VW. Association of Coffee Consumption With Total and Cause-Specific Mortality Among Nonwhite Populations. Ann Intern Med. 2017 Aug 15;167(4):228-235.
35. Sado J, Kitamura T, Kitamura Y, Sobue T, Nishino Y, Tanaka H, Nakayama T, Tsuji I, Ito H, Suzuki T, Katanoda K, Tominaga S; Three-Prefecture Cohort Study Group. Association between coffee consumption and all-sites cancer incidence and mortality. Cancer Sci. 2017 Oct;108(10):2079-2087.
36. Yan Y, Sui X, Yao B, Lavie CJ, Blair SN. Is There a Dose-Response Relationship between Tea Consumption and All-Cause, CVD, and Cancer Mortality? J Am Coll Nutr. 2017 May-Jun;36(4):281-286.
37. Zhao LG, Li HL, Sun JW, Yang Y, Ma X, Shu XO, Zheng W, Xiang YB. Green tea consumption and cause-specific mortality: Results from two prospective cohort studies in China. J Epidemiol. 2017 Jan;27(1):36-41.
38. Navarro AM, Martinez-Gonzalez MÁ, Gea A, Grosso G, Martín-Moreno JM, Lopez-Garcia E, Martin-Calvo N, Toledo E. Coffee consumption and total mortality in a Mediterranean prospective cohort. Am J Clin Nutr. 2018 Nov 1;108(5):1113-1120.
39. van den Brandt PA. Coffee or Tea? A prospective cohort study on the associations of coffee and tea intake with overall and cause-specific mortality in men versus women. Eur J Epidemiol. 2018 Feb;33(2):183-200.
40. Abe SK, Saito E, Sawada N, Tsugane S, Ito H, Lin Y, Tamakoshi A, Sado J, Kitamura Y, Sugawara Y, Tsuji I, Nagata C, Sadakane A, Shimazu T, Mizoue T, Matsuo K, Naito M, Tanaka K, Inoue M; Research Group for the Development and Evaluation of Cancer Prevention Strategies in Japan. Coffee consumption and mortality in Japanese men and women: A pooled analysis of eight population-based cohort studies in Japan (Japan Cohort Consortium). Prev Med. 2019 Jun;123:270-277.
41. Torres-Collado L, Garcia-de-la-Hera M, Navarrete-Muñoz EM, Notario-Barandiaran L, Gonzalez-Palacios S, Zurriaga O, Melchor I, Vioque J. Coffee consumption and mortality from all causes of death, cardiovascular disease and cancer in an elderly Spanish population. Eur J Nutr. 2019 Sep;58(6):2439-2448.
42. Yamakawa M, Wada K, Goto Y, Mizuta F, Koda S, Uji T, Nagata C. Associations between coffee consumption and all-cause and cause-specific mortality in a Japanese city: the Takayama study. Public Health Nutr. 2019 Oct;22(14):2561-2568.
43. Lukic M, Barnung RB, Skeie G, Olsen KS, Braaten T. Coffee consumption and overall and cause-specific mortality: the Norwegian Women and Cancer Study (NOWAC). Eur J Epidemiol. 2020 Oct;35(10):913-924.
44. Kim SA, Tan LJ, Shin S. Coffee Consumption and the Risk of All-Cause and Cause-Specific Mortality in the Korean Population. J Acad Nutr Diet. 2021 Nov;121(11):2221-2232.e4.
45. Sakamaki T, Kayaba K, Kotani K, Namekawa M, Hamaguchi T, Nakaya N, Ishikawa S. Coffee consumption and mortality in Japan with 18 years of follow-up: the Jichi Medical School Cohort Study. Public Health. 2021 Feb;191:23-30.
46. Torres-Collado L, Compañ-Gabucio LM, González-Palacios S, Notario-Barandiaran L, Oncina-Cánovas A, Vioque J, García-de la Hera M. Coffee Consumption and All-Cause, Cardiovascular, and Cancer Mortality in an Adult Mediterranean Population. Nutrients. 2021 Apr 9;13(4):1241.
47. Chieng D, Canovas R, Segan L, Sugumar H, Voskoboinik A, Prabhu S, Ling LH, Lee G, Morton JB, Kaye DM, Kalman JM, Kistler PM. The impact of coffee subtypes on incident cardiovascular disease, arrhythmias, and mortality: long-term outcomes from the UK Biobank. Eur J Prev Cardiol. 2022 Dec 7;29(17):2240-2249.
48. Inoue-Choi M, Ramirez Y, Freedman ND, Loftfield E. Tea Consumption and All-Cause and Cause-Specific Mortality in the UK Biobank. Ann Intern Med. 2023 Feb;176(2):eL220478.
49. Shin S, Lee JE, Loftfield E, Shu XO, Abe SK, Rahman MS, Saito E, Islam MR, Tsugane S, Sawada N, Tsuji I, Kanemura S, Sugawara Y, Tomata Y, Sadakane A, Ozasa K, Oze I, Ito H, Shin MH, Ahn YO, Park SK, Shin A, Xiang YB, Cai H, Koh WP, Yuan JM, Yoo KY, Chia KS, Boffetta P, Ahsan H, Zheng W, Inoue M, Kang D, Potter JD, Matsuo K, Qiao YL, Rothman N, Sinha R. Coffee and tea consumption and mortality from all causes, cardiovascular disease and cancer: a pooled analysis of prospective studies from the Asia Cohort Consortium. Int J Epidemiol. 2022 May 9;51(2):626-640.
50. Liu D, Li ZH, Shen D, Zhang PD, Song WQ, Zhang WT, Huang QM, Chen PL, Zhang XR, Mao C. Association of Sugar-Sweetened, Artificially Sweetened, and Unsweetened Coffee Consumption With All-Cause and Cause-Specific Mortality : A Large Prospective Cohort Study. Ann Intern Med. 2022 Jul;175(7):909-917.
51. Treskes RW, Clausen J, Marott JL, Jensen GB, Holtermann A, Gyntelberg F, Jensen MT. Use of sugar in coffee and tea and long-term risk of mortality in older adult Danish men: 32 years of follow-up from a prospective cohort study. PLoS One. 2023 Oct 18;18(10):e0292882.
52. Hu Y, Yang L, He J. Physical activity combined with tea consumption could further reduce all-cause and cancer-specific mortality. Sci Rep. 2024;14(1):23535.
53. Yang R, Lei Q, Liu Z, Shan X, Han S, Tang Y, Niu F, Liu H, Jiang W, Wei W, Han T. Relationship between timing of coffee and tea consumption with mortality (total, cardiovascular disease and diabetes) in people with diabetes: the U.S. National Health and Nutrition Examination Survey, 2003-2014. BMC Med. 2024;22(1):526.
54. Amani-Beni R, Sadeghi M, Nouri F, Darouei B, Mohammadifard N, Boshtam M, Hosseinkhani R, Sarrafzadegan N. Tea and coffee consumption and the 15-Year risk of cardiovascular events: the Isfahan cohort study (ICS). Nutr J. 2025;24(1):30.

*The study was performed in the same study population as the included study*

1. Loftfield E, Cornelis MC, Caporaso N, Yu K, Sinha R, Freedman N. Association of Coffee Drinking With Mortality by Genetic Variation in Caffeine Metabolism: Findings From the UK Biobank. JAMA Intern Med. 2018;178(8):1086-1097.
2. Zhou H, Nie J, Cao Y, Diao L, Zhang X, Li J, Chen S, Zhang X, Chen G, Zhang Z, Li B. Association of daily sitting time and coffee consumption with the risk of all-cause and cardiovascular disease mortality among US adults. BMC Public Health. 2024;24(1):1069.

**Table S2** Characteristics of the included studies on the association between coffee or tea consumption and risk of CVD and all-cause and cause-specific mortality in individuals with diabetes mellitus

| Reference | Cohort/data source | Sample size | Sex | Mean age, years | Type of DM | Follow-up, years | Exposure, exposure identification | Outcomes (n) | Adjustments |
| --- | --- | --- | --- | --- | --- | --- | --- | --- | --- |
| Sesso et al,. 2003 [18] | The College Alumni Health Study, USA | 626 | Both | 59.5 | Any DM | 15 | Tea, FFQ | CVD incidence (216) | Age, sex, BMI, physical activity, hypertension, smoking status, alcohol consumption, and early parental death |
| Bidel et al.,  2006 [19] | The North Karelia Project, Finland | 3,837 | Both | 49.5 | T2DM | 20.8 | Coffee, FFQ | All-cause mortality (1,471)  CVD mortality (909)  CHD mortality (598) | Age, sex, study year, BMI, systolic blood pressure, total cholesterol, education, alcohol, tea consumption, and smoking status |
| Zhang et al. 2009 [20] | The Nurses’ Health Study, USA | 7,170 | Women | 42.5 | T2DM | 8.74 | Coffee (decaffeinated and caffeinated), FFQ | All-cause mortality (734)  CHD mortality (217)  CVD incidence (658)  CHD incidence (434)  Stroke incidence (224) | Age, smoking status, BMI, alcohol intake, parental history of MI, history of hypertension, hyper-cholesterolemia, menopausal status and use of hormone therapy, physical activities, multivitamin use and vitamin E supplement use, total energy intake, duration of diabetes, and hypoglycemic medication, polyunsaturated, saturated, and trans fat, n-3, glycemic load, dietary cereal fiber and folate intake. |
| Zhang et al. 2009 [21] | The Health Professionals Follow-up Study USA | 3,497 | Men | 57.5 | T2DM | 6.95 | Coffee (decaffeinated and caffeinated), FFQ | All-cause mortality (538)  CVD incidence (435)  CHD incidence (324)  Stroke incidence (111) | Age, smoking status, BMI, alcohol intake, parental history of MI, history of hypertension, hypercholesterolemia, menopausal status and use of hormone therapy, physical activities, multivitamin use and vitamin E supplement use, total energy intake, duration of diabetes, and hypoglycemic medication, polyunsaturated, saturated, and trans fat, n-3, glycemic load, dietary cereal fiber and folate intake. |
| Freedman et al.,  2012 [22] | The National Institutes of Health–AARP Diet and Health Study, USA | 24,174 | Both | 60.5 | Any DM | 13.6 | Coffee, FFQ | All-cause mortality (7,634) | Age; body-mass index; race or ethnic group; level of education; alcohol consumption; the number of cigarettes smoked per day, use or nonuse of pipes or cigars, and time of smoking cessation; health status; marital status; physical activity; total energy intake; consumption of fruits, vegetables, red meat, white meat, and saturated fat; and use or nonuse of vitamin supplements. |
| Loftfield et al.,  2015 [23] | The Prostate, Lung, Colorectal, and Ovarian (PLCO) Cancer Screening Trial, USA | 3,838 | Both | 64.5 | Any DM | 9 | Coffee, FFQ | All-cause mortality (173) | Age, sex, detailed smoking history, race/ethnicity, educational level, marital status, employment status, body mass index, any supplemental vitamin use in the previous 12 months, regular ibuprofen use in the previous 12 months, regular aspirin use in the previous 12 months, receipt of menopausal hormone therapy, alcohol consumption, total daily energy intake, and quintile of daily intake of red and processed meat, white meat, saturated fat, fruits, and vegetables |
| Saito et al.,  2015 [24] | Japan Public Health Center-Based Prospective Study, Japan | 3,936 | Both | 54.5 | Any DM | 18.7 | Coffee, FFQ | All-cause mortality (1,203) | Age; public health center area; alcohol consumption; BMI; history of hypertension; history of diabetes; leisure-time sports or physical exercise; intakes of green tea, Chinese tea, black tea, soda and juice, energy, fruit, vegetables, fish, meat, dairy products, rice, and miso soup; and job status. |
| van Dongen et al., 2017 [25] | The Alpha Omega Cohort, Netherlands | 884 | Both | 69 | Any DM | 7.1 | Coffee, FFQ | All-cause mortality (240)  CVD mortality (108)  CHD mortality (68) | Age, sex, treatment code, BMI, physical activity, educational level, smoking status, alcohol use, and intake of total energy, tea, whole grains, red/processed meats, dairy, vegetables/fruits, chocolate, sugar-sweetened beverages, plant oils, legumes and nuts/seeds. |
| Neves et al. 2018 [26] | The National Health and Nutrition Examination Survey, USA | 3,948 | Both | 58.6 | Any DM | 4.75 | Coffee, single 24-h dietary recall | All-cause mortality (768)  CVD mortality (199)  Cancer mortality (127) | Age, race, annual family income, smoking status, and diabetic kidney disease, body mass index, education level, daily carbohydrate consumption, alcohol consumption, years since diabetes diagnosis, diagnosis of hypertension, retinopathy, macrovascular complications, insulin treatment and survey cycle |
| Komorita et al.,  2020 [27] | The Fukuoka Diabetes Registry, Japan | 4,923 | Both | 66 | T2DM | 5.3 | Coffee and green tea, FFQ | All-cause mortality (309)  CVD mortality (76)  Cancer mortality (114) | Age, sex, BMI, diabetes duration, current smoking habit, current alcohol intake, LTPA, sleep duration, HbA1c, UACR, systolic blood pressure, LDL cholesterol, history of CVD and cancer |
| Wang et al. 2020 [28] | The China-PAR project, China | 5,169 | Both | 51.2 | Any DM | 7.3 | Tea, FFQ | All-cause mortality (590) | Age, sex, region, area, cohort, education level , family history of ASCVD, smoking, drinking, physical activity level, dietary factors, body mass index, systolic blood pressure, fasting blood glucose, total cholesterol, high-density lipoprotein-cholesterol |
| Nie et al. 2021 [29] | The China Kadoorie Biobank, China | 30,300 (all-cause mortality and CVD mortality); 26,162 (CHD incidence and stroke incidence) | Both | 58.2 | Any DM | 10.6 | Tea, FFQ | All-cause mortality (6,572)  CVD mortality (2,117)  CHD incidence (4,895)  Stroke incidence (5,759) | Age; education; random glucose; treatment for diabetes; smoking; alcohol intake; level of physical activity; intakes of red meat, fresh vegetables, and fruits; BMI; waist circumference; and baseline prevalence of hypertension, cancer, stroke, and coronary heart disease. |
| Ruggiero et al. 2021 [30] | The Moli-sani Study, Italy | 816 | Both | 55.3 | Any DM | 8.3 | Coffee, FFQ | All-cause mortality (113) | Age, sex, energy intake, educational level, residence, smoking, number of cigarettes per day, abdominal obesity, leisure-time physical activity, diabetes, hypertension, hyperlipidaemia, adherence to Mediterranean diet, tea intake, added sugar |
| Chen et al. 2022 [31] | The United Kingdom Biobank, UK | 26367 | Both | 58.6 | Any DM | 12.1 | Coffee and tea, FFQ | All-cause mortality (NR)  CVD mortality (NR) | Age, sex, ethnicity, education levels, BMI, smoking status, alcohol intake frequency, physical activity, dietary pattern, general health status, hypertension, and depression. |
| Ma et al. 2023 [32] | The Nurses’ Health Study and Health Professionals Follow-up Study, USA | 15,486 | Both | 61.3 | T2DM | 18.5 | Coffee and tea, FFQ | All-cause mortality (7,638)  CVD mortality (2,397)  Cancer mortality (1,433)  CVD incidence (3,447) | Age, duration of diabetes, sex, white ethnicity, physical activity, smoking status, alcohol consumption, menopausal status and post-menopausal hormone use, family history of type 2 diabetes or myocardial infarction, intake of total energy, the modified Alternative Healthy Eating Index score, history of hypertension or hypercholesterolemia, use of antihypertensive or lipid-lowering drug, aspirin use, diabetes drug use, and change in body mass index before to after diabetes diagnosis |
| Liu et al. 2023 [33] | The United Kingdom Biobank, UK | 9,964 | Both | 57.9 | T2DM | 12.7 | Coffee, FFQ | CVD incidence (1,860 )  CHD incidence (1,411)  Stroke incidence (329) | Age, sex, ethnicity, Townsend deprivation index, BMI, physical activity, alcohol consumption, diet score, hypertension (yes, no), hyperlipidemia (yes, no), HbA1c levels (continuous, mmol/mol), and diabetes duration (continuous, years). |
| Wang et al. 2025 [34] | Comprehensive Research on the Prevention and Control of Diabetes, China | 15,718 | Both | 61.98 | T2DM | 9.77 | Tea, FFQ | All-cause mortality (3,046)  CVD mortality (922)  CHD mortality (159)  Cancer mortality (736) | Age, sex, educational level , marital status , annual household income , smoking status , alcohol drinking status , body mass index (kg/m2), total physical activity (MET‐h/day), duration of diabetes (years), oral antidiabetic medication use (no, yes), insulin use (no, yes), fruit consumption , vegetable consumption , animal meat consumption. |

**Table S3** Quality assessment for studies included the meta-analysis the association between coffee or tea consumption and risk of cardiovascular disease and all-cause and cause-specific mortality in individuals with diabetes mellitus (Newcastle Ottawa Scale).

| Author, year | Selection | | | | Comparability | Outcome/ Exposure | | | Total Score |
| --- | --- | --- | --- | --- | --- | --- | --- | --- | --- |
|  | Representativeness of the exposed cohort | Selection of the non-exposed cohort | Ascertainment of exposure^1^ | Outcome of interest was not present at start of study | Control for additional factors^2^ | Assessment of outcome | Follow-up long enough^4^ | Adequacy of follow-up of cohorts^5^ |  |
| Sesso, 2003 (18) | - | * | - | * | ** | * | * | - | 6 |
| Bidel, 2006 (19) | * | * | - | * | ** | * | * | - | 7 |
| Zhang, 2009 (20) | - | * | * | * | ** | * | - | - | 6 |
| Zhang, 2009 (21) | - | * | * | * | ** | * | - | - | 6 |
| Freedman, 2012 (22) | - | * | * | * | ** | * | * | - | 7 |
| Saito, 2015 (23) | * | * | * | * | ** | * | * | - | 8 |
| Loftfield, 2015 (24) | - | * | * | * | ** | * | - | - | 6 |
| van Dongen, 2017 (25) | - | * | * | * | ** | * | - | - | 6 |
| Neves, 2018 (26) | * | * | * | * | ** | * | - | - | 7 |
| Komorita, 2020 (27) | * | * | * | * | ** | * | - | * | 8 |
| Wang, 2020 (28) | * | * | - | * | ** | * | - | - | 6 |
| Nie, 2021 (29) | * | * | * | * | ** | * | * | - | 8 |
| Ruggiero, 2021 (30) | * | * | * | * | ** | * | - | - | 7 |
| Chen, 2022 (31) | * | * | * | * | ** | * | * | - | 8 |
| Ma, 2023 (32) | * | * | * | * | ** | * | * | - | 8 |
| Liu, 2023 (33) | * | * | * | * | ** | * | * | - | 8 |
| Wang, 2025 (34) | * | * | - | * | ** | * | - | * | 7 |

^1^One star if the study used dietary assessment method that has been validated.

^2^For age and sex adjusted: one star. Second star for additional adjustments for three of the following variables: body mass index, alcohol drinking, physical activity, smoking status, and energy intake.

^3^At least 10 years.

^4^At least 80%.

^5^0-3: low quality, 4-6: moderate quality, 7-9: high quality.

| **Table S4** The quality of evidence for the association between coffee or tea consumption and risk of cardiovascular disease and all-cause and cause-specific mortality in individuals with diabetes mellitus.   \|  \| \| Risk of bias, study quality, and study limitations \| Precision \| Heterogeneity \| Directness \| Publication bias \| Funding bias \| Effect size \| Dose-response \| Total score \| Quality of evidence \| \| --- \| --- \| --- \| --- \| --- \| --- \| --- \| --- \| --- \| --- \| --- \| --- \| \| Tea \| \|  \|  \|  \|  \|  \|  \|  \|  \|  \|  \| \|  \| All-cause mortality \| 2 \| 1 \| 0.4 \| 1 \| 0.5 \| 1 \| 0 \| 0 \| 5.9 \| Low \| \|  \| CVD mortality \| 2 \| 1 \| 0 \| 1 \| 0 \| 1 \| 0 \| 1 \| 6 \| Moderate \| \|  \| Cancer mortality \| 2 \| 0 \| 0 \| 1 \| 0 \| 1 \| 0 \| 0 \| 4 \| Low \| \|  \| CVD incidence \| 2 \| 1 \| 0 \| 1 \| 0 \| 1 \| 0 \| 0 \| 5 \| Low \| \| Coffee \| \|  \|  \|  \|  \|  \|  \|  \|  \|  \|  \| \|  \| All-cause mortality \| 2 \| 1 \| 0.4 \| 1 \| 0.5 \| 1 \| 0 \| 1 \| 6.9 \| Moderate \| \|  \| CVD mortality \| 2 \| 1 \| 0 \| 1 \| 0.5 \| 1 \| 0 \| 1 \| 6.5 \| Moderate \| \|  \| CHD mortality \| 1 \| 1 \| 0 \| 1 \| 0 \| 1 \| 1 \| 1 \| 6 \| Moderate \| \|  \| Cancer mortality \| 2 \| 0 \| 0 \| 1 \| 0 \| 1 \| 0 \| 0 \| 4 \| Low \| \|  \| CVD incidence \| 2 \| 1 \| 0 \| 1 \| 0 \| 1 \| 0 \| 1 \| 6 \| Moderate \| \|  \| CHD incidence \| 2 \| 1 \| 0 \| 1 \| 0 \| 1 \| 0 \| 1 \| 6 \| Moderate \| \|  \| Stroke incidence \| 2 \| 0 \| 0 \| 1 \| 0 \| 1 \| 0 \| 0 \| 4 \| Low \| |
| --- | --- | --- | --- | --- | --- | --- | --- | --- | --- | --- | --- | --- | --- | --- | --- | --- | --- | --- | --- | --- | --- | --- | --- | --- | --- | --- | --- | --- | --- | --- | --- | --- | --- | --- | --- | --- | --- | --- | --- | --- | --- | --- | --- | --- | --- | --- | --- | --- | --- | --- | --- | --- | --- | --- | --- | --- | --- | --- | --- | --- | --- | --- | --- | --- | --- | --- | --- | --- | --- | --- | --- | --- | --- | --- | --- | --- | --- | --- | --- | --- | --- | --- | --- | --- | --- | --- | --- | --- | --- | --- | --- | --- | --- | --- | --- | --- | --- | --- | --- | --- | --- | --- | --- | --- | --- | --- | --- | --- | --- | --- | --- | --- | --- | --- | --- | --- | --- | --- | --- | --- | --- | --- | --- | --- | --- | --- | --- | --- | --- | --- | --- | --- | --- | --- | --- | --- | --- | --- | --- | --- | --- | --- | --- | --- | --- | --- | --- | --- | --- | --- | --- | --- | --- | --- | --- | --- | --- | --- | --- | --- | --- | --- | --- | --- | --- | --- | --- | --- |

**Figure S2**. (A) Forest plot of the association between the highest versus lowest coffee consumption category and the risk of all-cause mortality in individuals with diabetes mellitus (18,112 deaths (number of deaths was not reported in one study (31))/87,393 participants); (B) Forest plot of the linear association between per cup increase in daily coffee consumption and the risk of all-cause mortality in individuals with diabetes mellitus (18,225 deaths /61,842 participants).


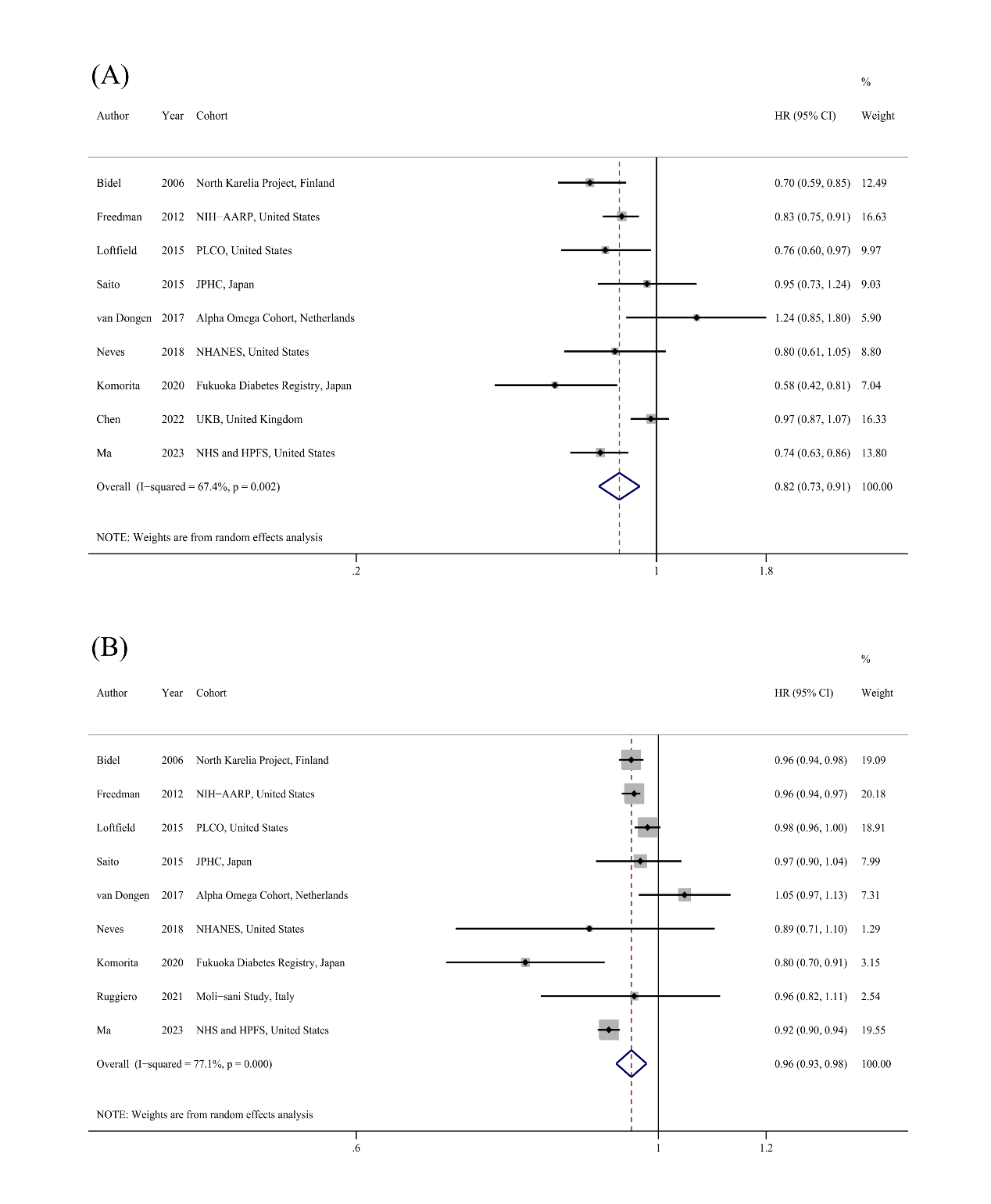


**Figure S3**. (A) Forest plot of the association between the highest versus lowest coffee consumption category and the risk of cardiovascular disease mortality in individuals people with diabetes mellitus (3,689 deaths (number of deaths was not reported in one study (31))/55,445 participants); (B) Forest plot of the linear association between per cup increase in daily coffee consumption and the risk of cardiovascular disease mortality in individuals with diabetes mellitus (3,802 deaths/29,894 participants).


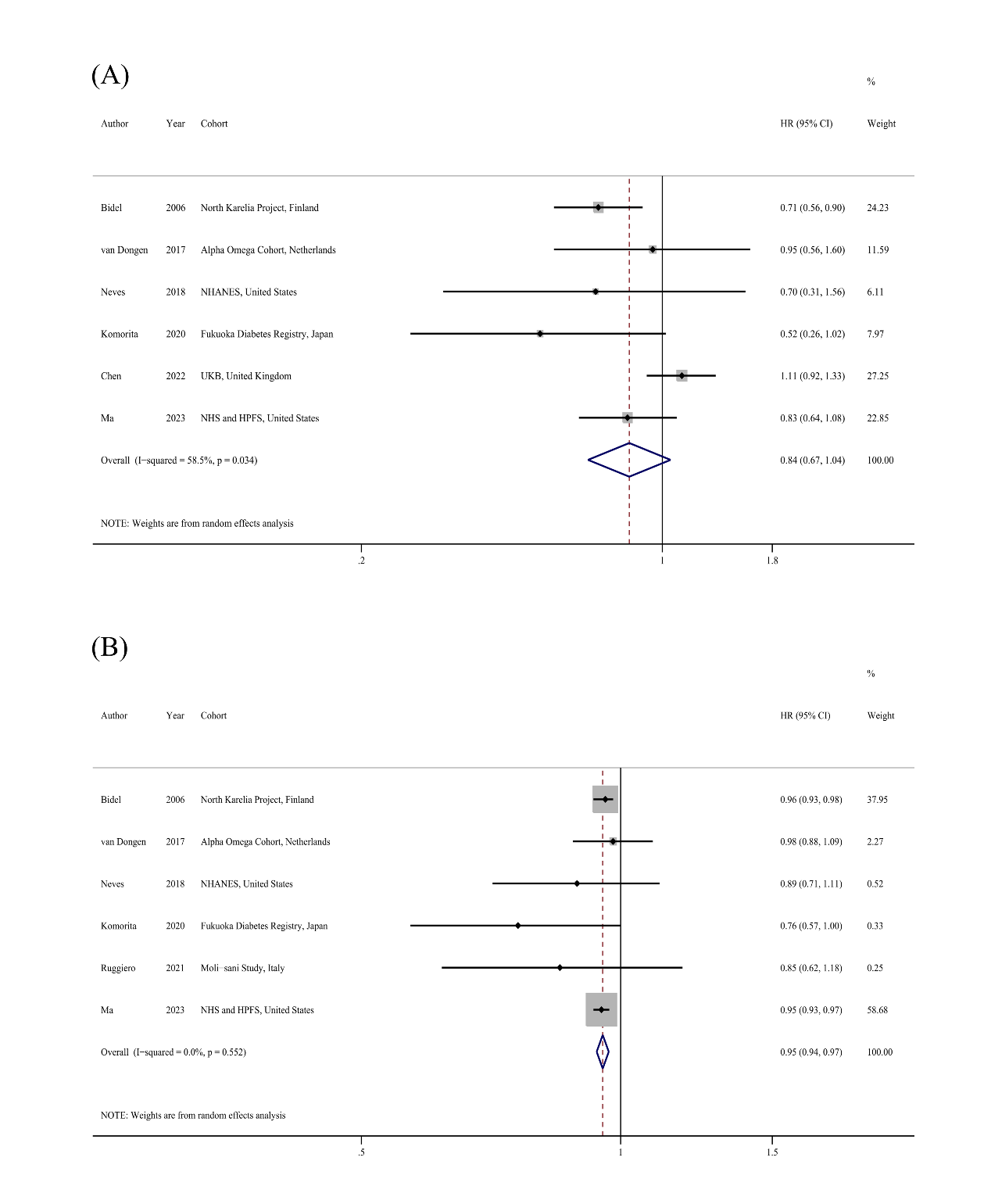


**Figure S4**. (A) Forest plot of the association between the highest versus lowest coffee consumption category and the risk of coronary heart disease mortality in individuals with diabetes mellitus (883 deaths/11,891 participants); (B) Forest plot of the linear association between per cup increase in daily coffee consumption and the risk of coronary heart disease mortality in individuals with diabetes mellitus (883 deaths/11,891 participants).


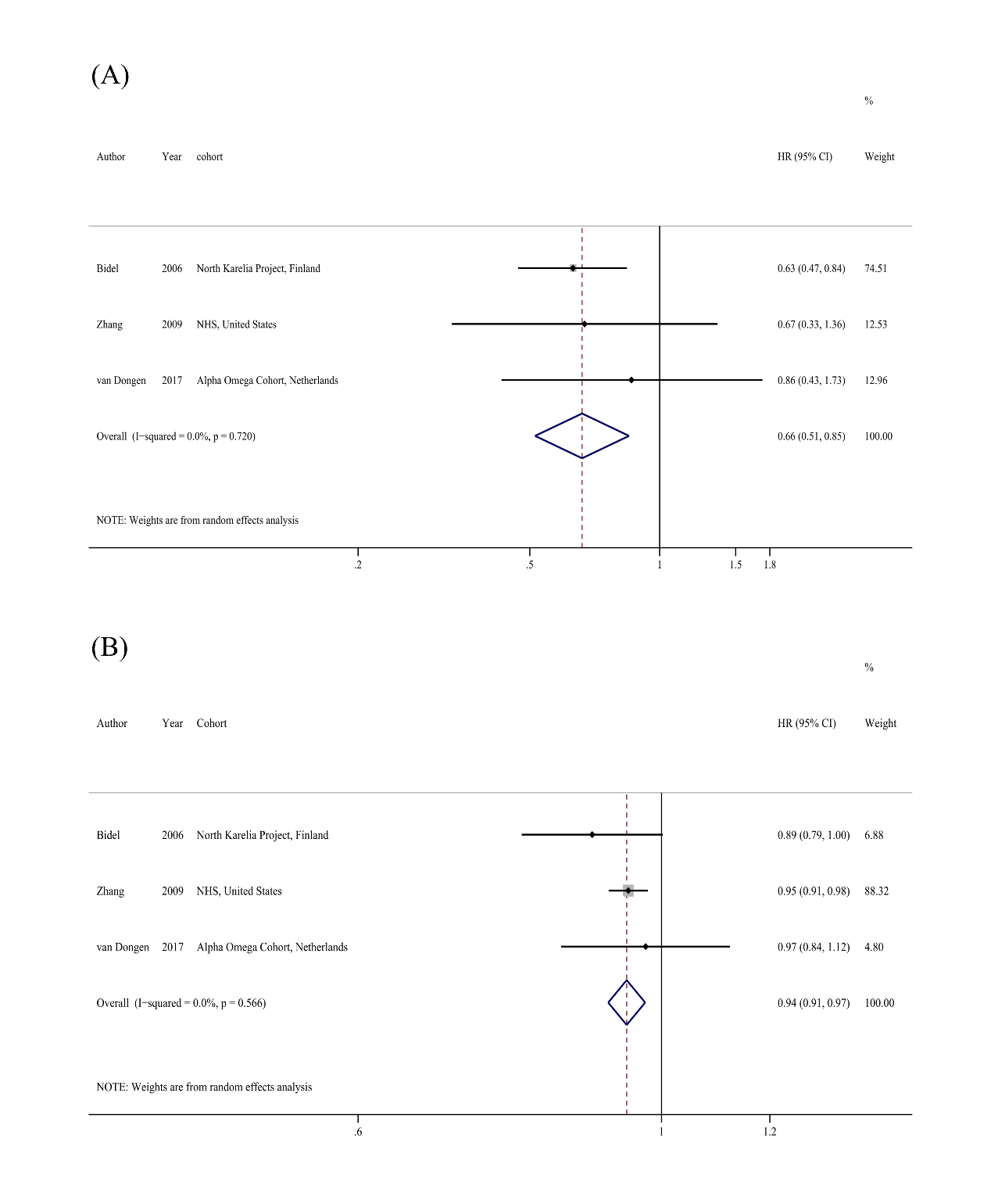


**Figure S5**. (A) Forest plot of the association between the highest versus lowest coffee consumption category and the risk of cancer mortality in individuals with diabetes mellitus (1,674 deaths/24,357 participants); (B) Forest plot of the linear association between per cup increase in daily coffee consumption and the risk of cancer mortality in individuals with diabetes mellitus (1,674 deaths/24,357 participants).


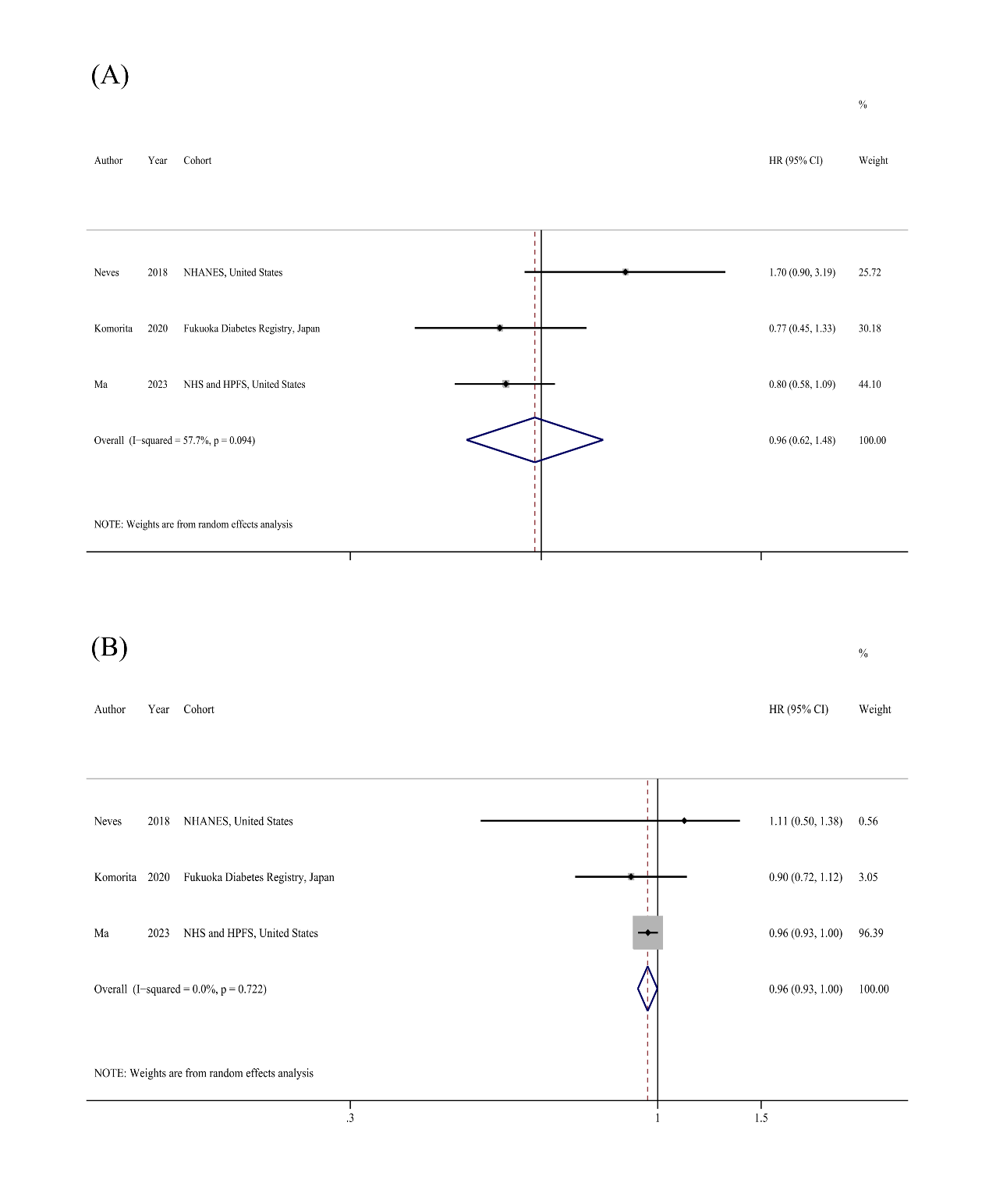


**Figure S6**. (A) Forest plot of the association between the highest versus lowest coffee consumption category and the risk of cardiovascular disease incidence in individuals with diabetes mellitus (5,307 cases/25,450 participants); (B) Forest plot of the linear association between per cup increase in daily coffee consumption and the risk of cardiovascular disease incidence in individuals with diabetes mellitus (5,307 cases/25,450 participants).


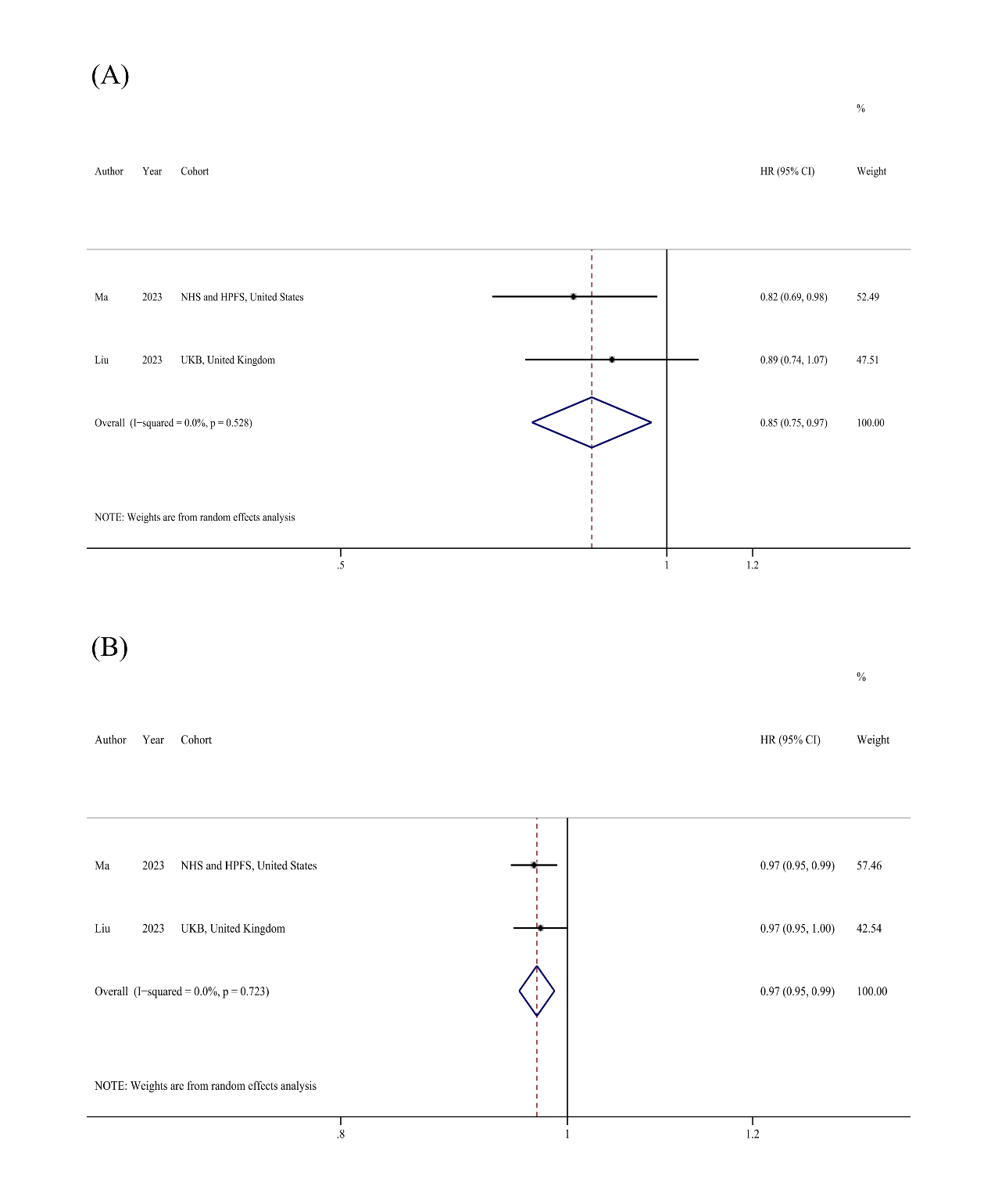


**Figure S7**. (A) Forest plot of the association between the highest versus lowest coffee consumption category and the risk of coronary heart disease incidence in individuals with diabetes mellitus (2,169 cases/20,631 participants); (B) Forest plot of the linear association between per cup increase in daily coffee consumption and the risk of coronary heart disease incidence in individuals with diabetes mellitus (2,169 cases/20,631 participants). .


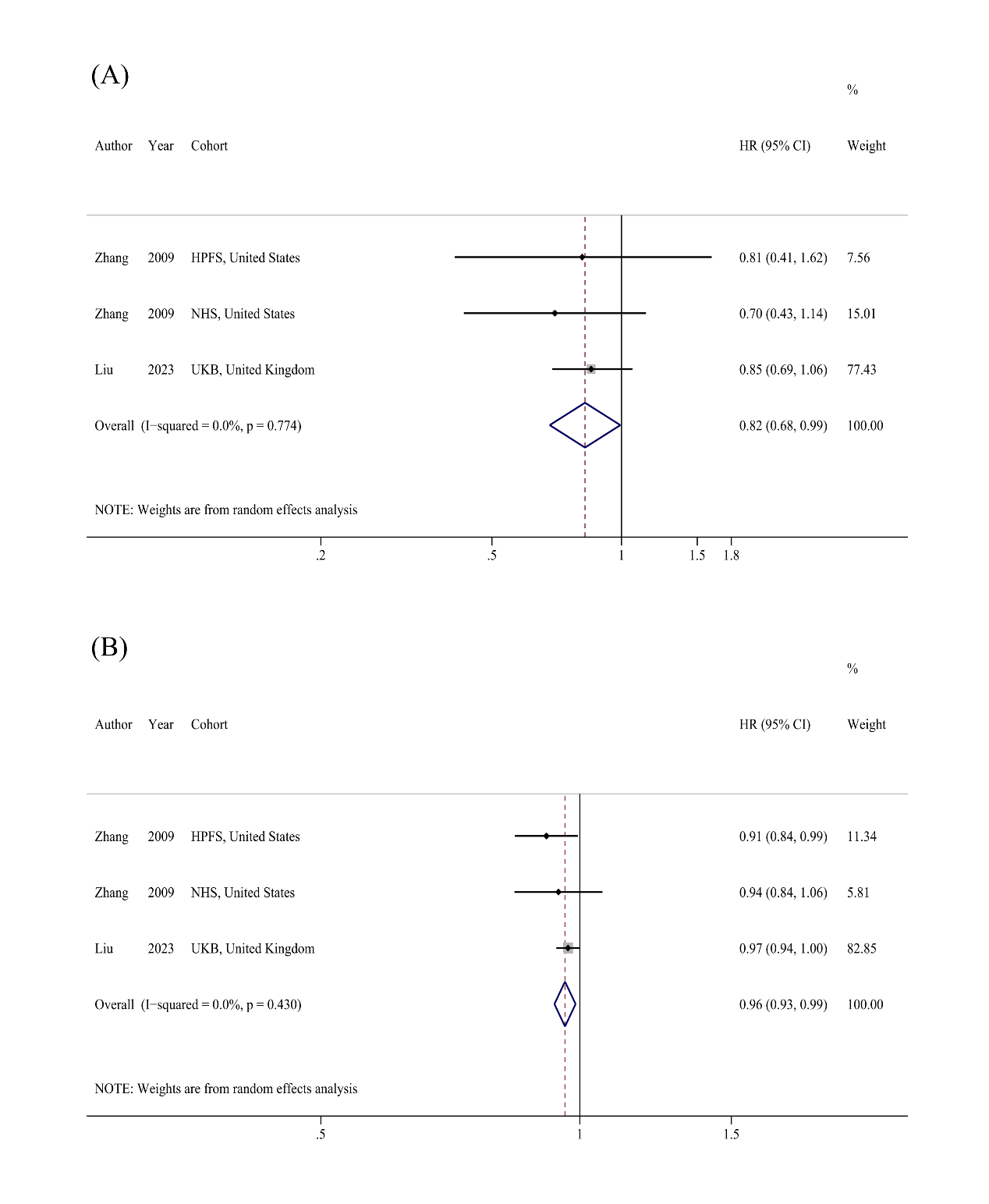


**Figure S8**. (A) Forest plot of the association between the highest versus lowest coffee consumption category and the risk of stroke incidence in individuals with diabetes mellitus (664 cases/20,631 participants); (B) Forest plot of the linear association between per cup increase in daily coffee consumption and the risk of stroke incidence in individuals with diabetes mellitus (664 cases/20,631 participants).


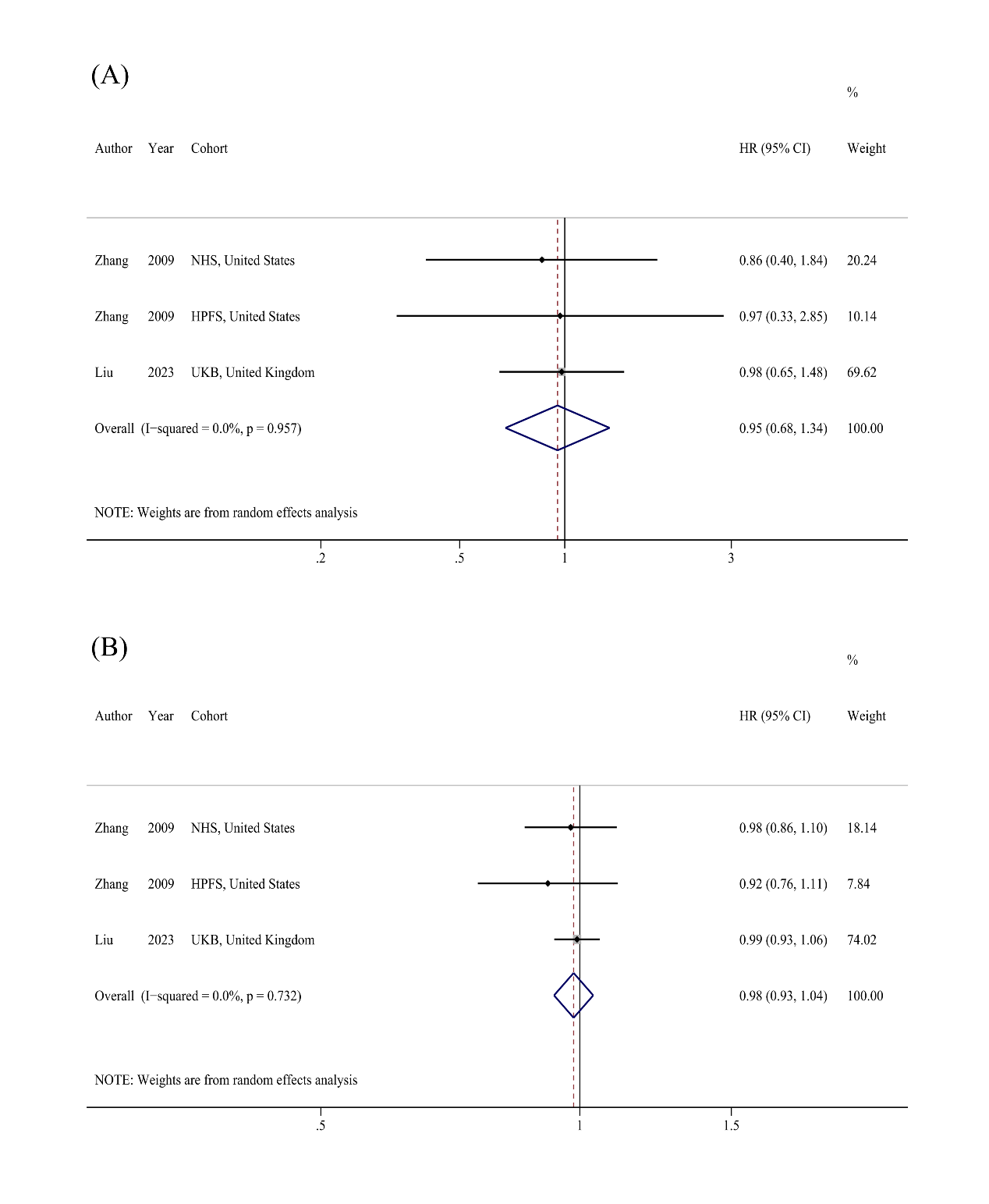


**Figure S9.** Forest plot of the association between coffee consumption and the risks of all-cause mortality, cardiovascular disease (CVD) mortality, coronary heart disease (CHD) mortality, cancer mortality, CVD incidence, CHD incidence, and stroke incidence in individuals with type 2 diabetes mellitus

**Figure S10**. (A) Forest plot of the association between the highest versus lowest tea consumption category and the risk of all-cause mortality in individuals with diabetes mellitus (18,155 cases (number of deaths was not reported in one study (31))/97,963 participants); (B) Forest plot of the linear association between per cup increase in daily tea consumption and the risk of all-cause mortality in individuals with diabetes mellitus (17,565 cases /66,427 participants).


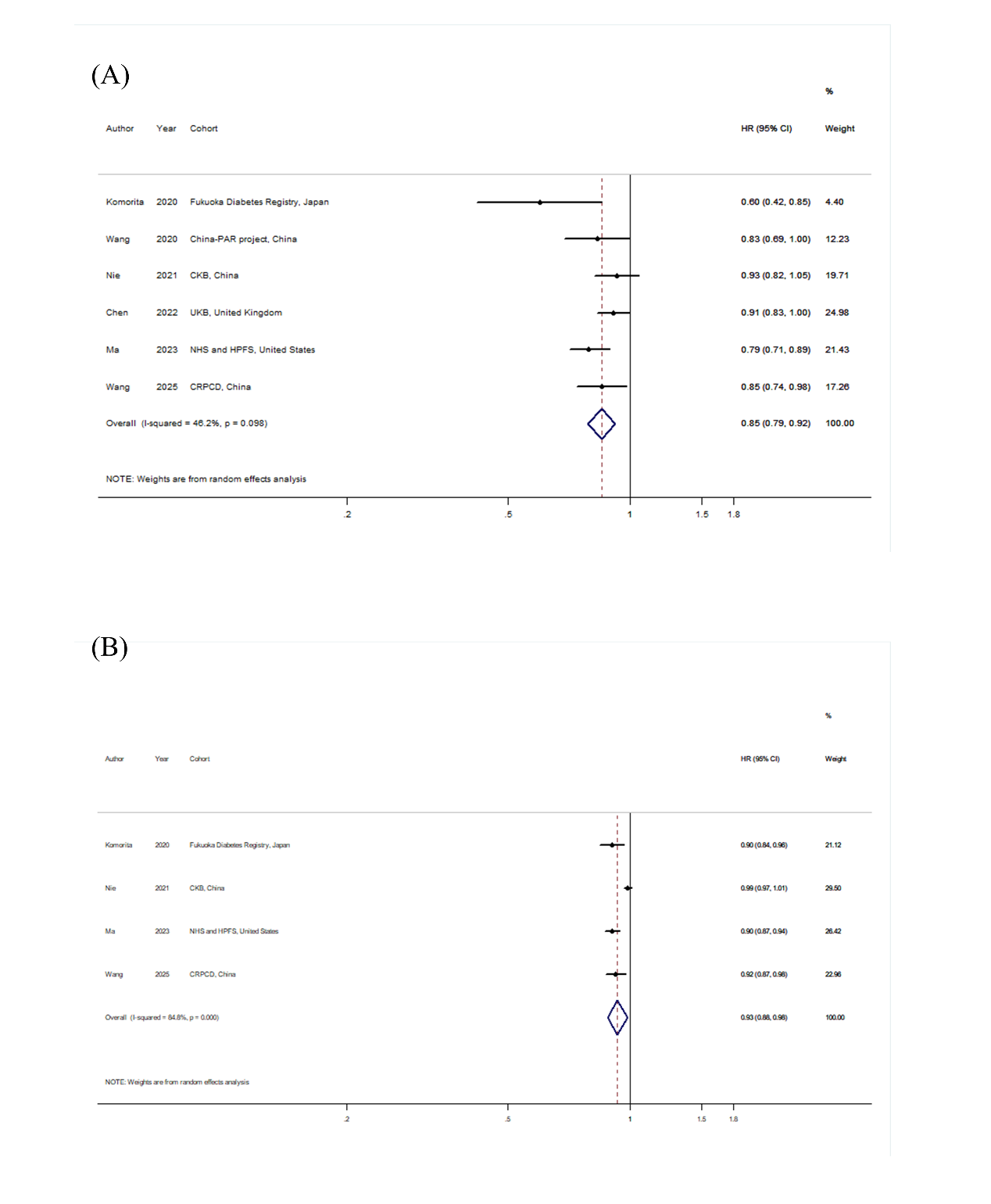


**Figure S11**. (A) Forest plot of the association between the highest versus lowest tea consumption category and the risk of CVD mortality in individuals with diabetes mellitus (5,512 cases (number of deaths was not reported in one study (31))/92,794 participants); (B) Forest plot of the linear association between per cup increase in daily tea consumption and the risk of CVD mortality in individuals with diabetes mellitus (3,395 cases /36,127 participants).


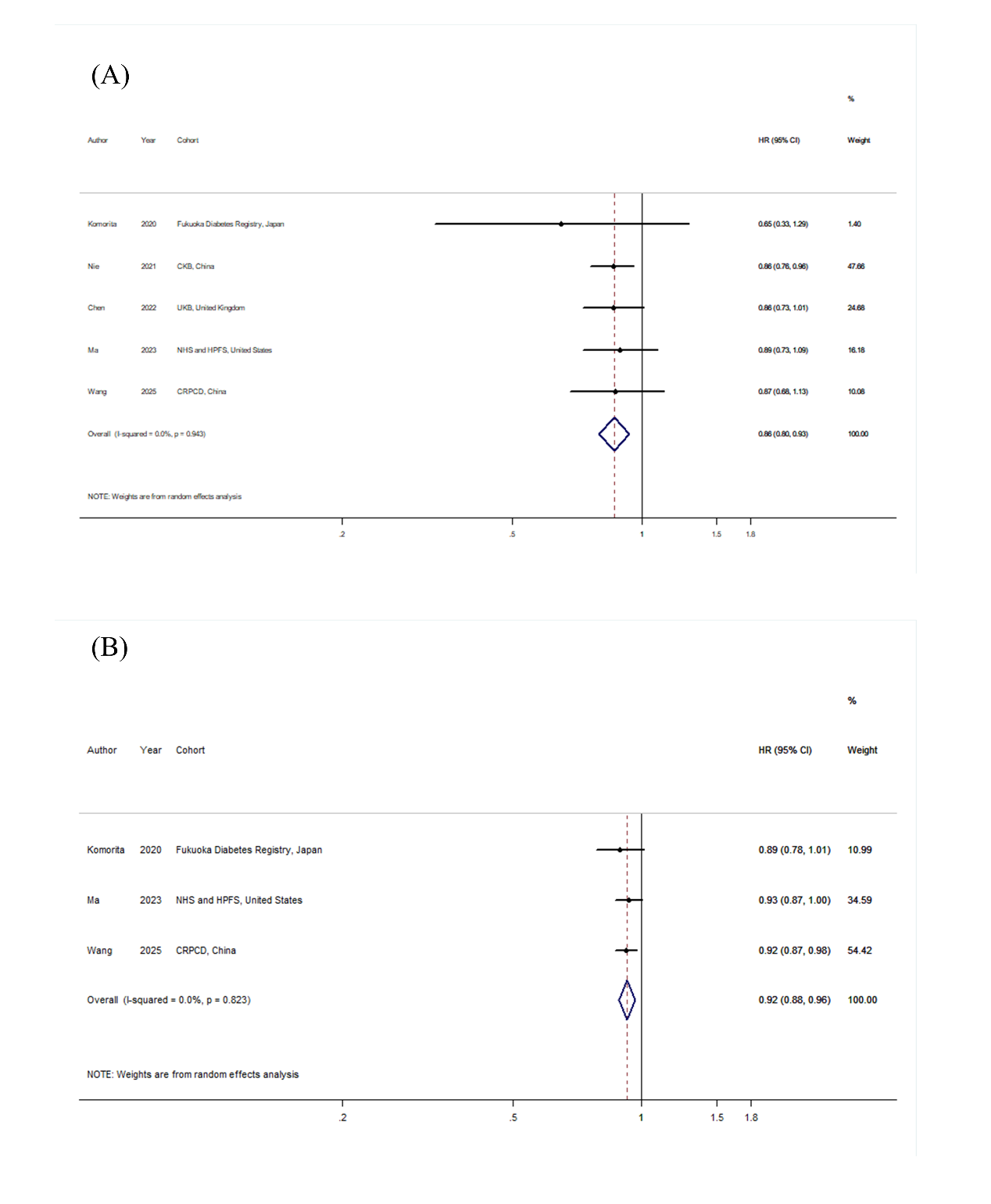


(B)

**Figure S12**. (A) Forest plot of the association between the highest versus lowest tea consumption category and the risk of cancer mortality in individuals with diabetes mellitus (2,283 cases/36,127 participants); (B) Forest plot of the linear association between per cup increase in daily tea consumption and the risk of cancer mortality in individuals with diabetes mellitus (2,283 cases /36,127 participants).


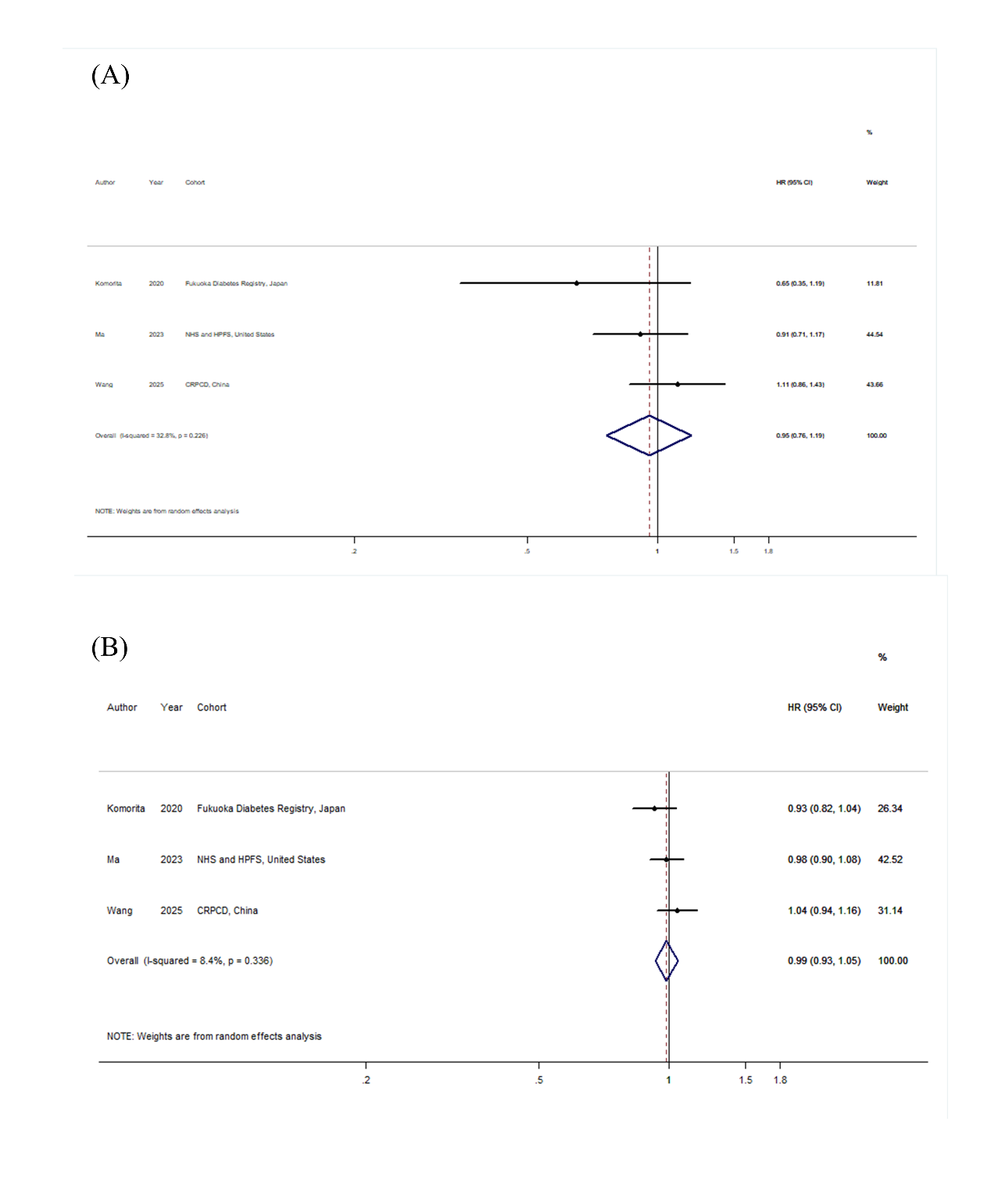


**Figure S13**. Forest plot of the association between the highest versus lowest tea consumption category and the risk of CVD incidence in individuals with diabetes mellitus (3,663 cases/16,112 participants)


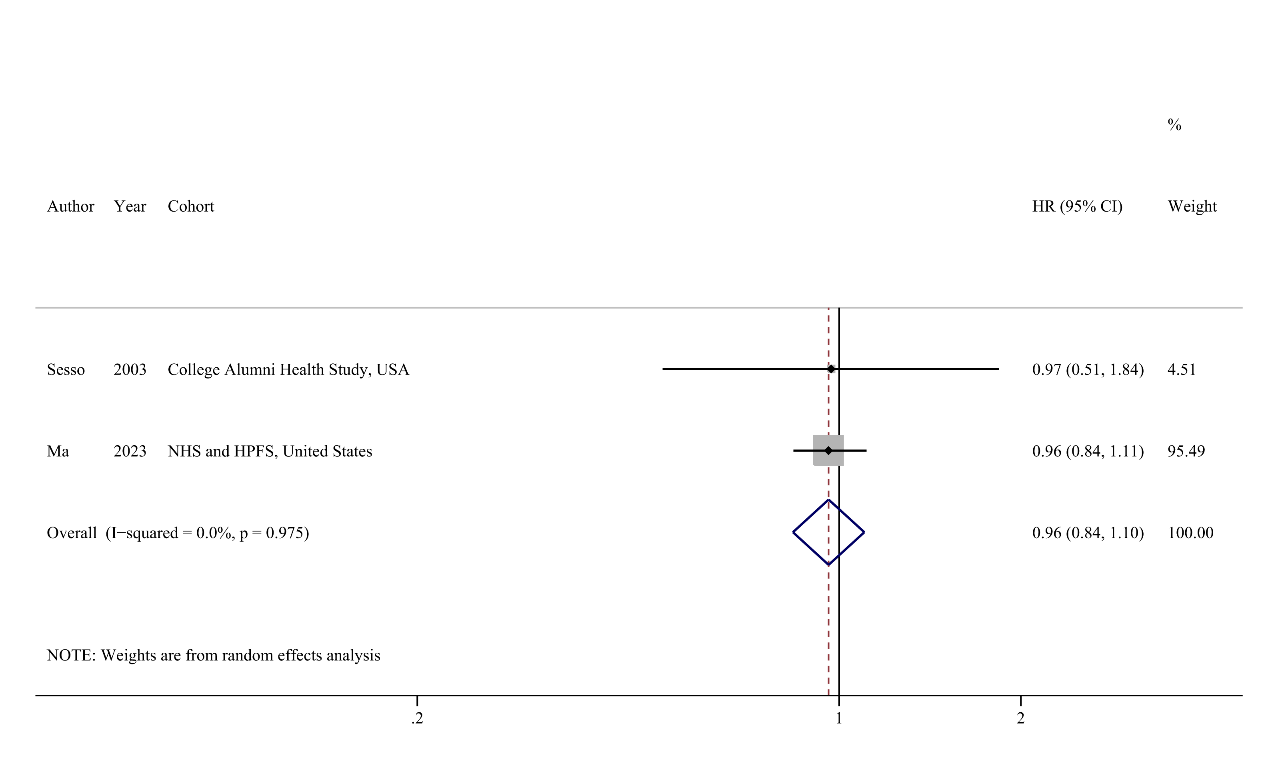


**Figure S14.** Forest plot of the association between tea consumption and the risks of all-cause mortality, cardiovascular disease (CVD) mortality, and cancer mortality in people with type 2 diabetes mellitus


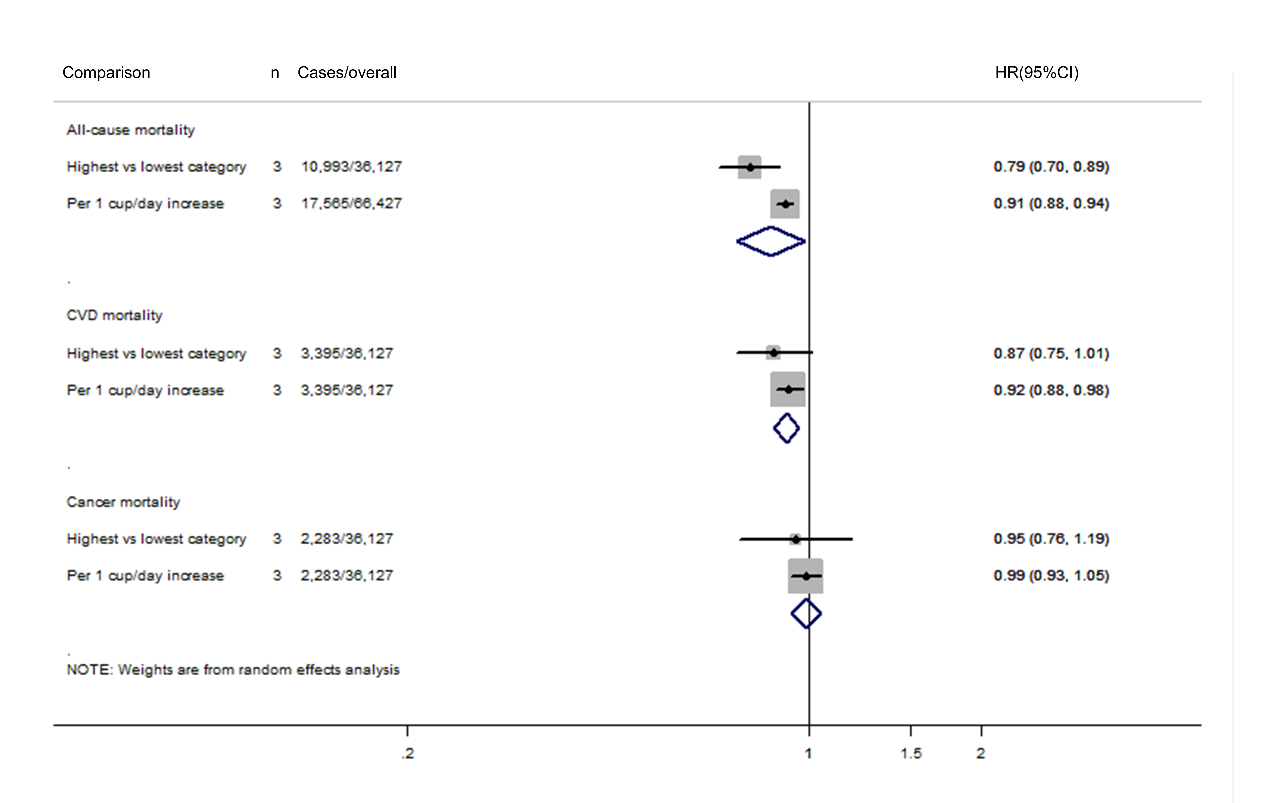

Supplement: Supplementary file 1 [file Table_1.docx]
